# Health questionnaire on back care knowledge and spine disease prevention for 6–10 years old children: development and psychometric evaluation

**DOI:** 10.1186/s12891-021-04667-x

**Published:** 2021-09-23

**Authors:** Brigitta Szilágyi, Péter Tardi, Borbála Magyar, Nóra Tanács-Gulyás, Fanny Romhányi, Elizabetta Vida, Alexandra Makai, Melinda Járomi

**Affiliations:** 1 Faculty of Health Sciences, University of Pécs, Doctoral School of Health Sciences, Vörösmarty street 4, Pécs, 7621 Hungary; 2 Faculty of Health Sciences, University of Pécs, Institute of Physiotherapy and Sport Science, Vörösmarty street 4, Pécs, 7621 Hungary

**Keywords:** Back care knowledge, Spine disease prevention, Child back school, Primary school children, Questionnaire validation

## Abstract

**Background:**

Back school programs, that improve back care and spine disease prevention knowledge are recommended at the age of 4-14 years. There is Health Questionnaire on Back Care Knowledge in the literature for children aged 14-17 years. At other ages, there is no questionnaire examining this knowledge. We aimed to develop a Health Questionnaire on Back Care and Spine Disease Prevention Knowledge for 6-10 years old children and validate its psychometric properties (internal consistency, test-retest reliability, agreement, convergent validity, discriminant validity) in 6-10 years old children, who attended back school program or not.

**Methods:**

463 children took part in the research (6-10 years old). The development was performed according to the Delphi method. The final version contained 7 questions. 463 participants completed the questionnaire twice with an interval of 7 days to evaluate test-retest reliability. The internal consistency was tested by Cronbach’s alpha value, test–retest reliability was calculated by Intraclass Correlation Coefficients (ICC), Standard Error of Measurement (SEM) and 95% of Minimal Detectable Change (MDC95) and Bland–Altman plots. Convergent validity was tested against the age variable and discriminant validity was tested by Kruskal-Wallis tests among the different subgroups.

**Results:**

Cronbach’s alpha of the total score was (α=0.797), showed a strong internal consistency with minimal SEM (0.606) and MDC95 (1.680). The test-retest result for the total score was strong (0.989), for the questions showed moderate to strong results (0.742-0.975), the limits of agreement of the Bland-Altman plot showed a narrow error of measurement range (-3.49-1.29), and the value of mean differences was −1.10 (SD ± 1.22). The convergent validity showed a weak, but significant relationship between total score and age (R=0.171; *p* < 0.001). The discriminant validity showed significantly different mean scores in non-back school and back school groups.

**Conclusion:**

For the examination of back care and spine disease prevention knowledge of 6-10 years old children, the questionnaire proved to be a valid and reliable tool. The knowledge requested in the questionnaire covers the knowledge material of the theoretical part of the back school for children aged 4-10 years.

**Supplementary Information:**

The online version contains supplementary material available at 10.1186/s12891-021-04667-x.

## Background

Back pain, but most of all, low back pain (LBP) shows an increasing prevalence among school-age youth [1]. This age group is exposed to a variety of risk factors during daily activity. Such intrinsic risk factors may include personal characteristics (gender, age, height, weight, etc.), functional status (curvature of the spine, asymmetries, inadequate flexibility, muscle strength, etc.), lifestyle (sedentary lifestyle, lack of physical activity, inadequate exercising, etc.) or even psychosocial factors (self-image, somatic symptoms, beliefs, disability, etc.) and extrinsic factors can be risk factors associated with the school environment (improperly designed school environment, furniture, long-term sitting, improper sitting position, school bag carrying, and its overload, etc.) [2, 3]. It is estimated that 60–80% of people experience LBP throughout their lives [4, 5]. LBP has already become a growing and serious public health problem among children and adolescents. The prevalence of LBP in children and adolescents is estimated to be very high, ranging from 7 to 72%, with an average of 39.9% [3–5]. Hestbaek et al. conducted an eight-year prospective study of LBP development from adolescence to adulthood, the results indicated that the presence of LBP among adolescents, a significant risk factor for the development of LBP among adults [6, 7]. This high prevalence of back pain implies a high social and economic charge and restrains a considerable part of the population in their personal psychosocial and functional life [2].

A frequently used method of prevention is the back school program (BSP) [8], applied in numerous countries [9–16]. Nowadays BSP education occurs in a rehabilitation setting and is directed at adults suffering the consequences of years of bad habits, including ineffective lifting patterns [17]. Sheldon recommends using BPS principles in early childhood as a proactive method of prevention of back pain, it should begin proactively in the elementary schools where it could reach the greatest number of people [18]. According to Geldhof, primary prevention should focus on good back functioning, instead of being focused on back pain prevalence [2].

Our habits determine our health, as does disease-specific knowledge. If we get to know the background of something, and we are aware of it, it is easier to develop new, good habits instead of bad ones. In the improvement of posture habits, the first is the habits [19], besides, the development of back care knowledge is important, which can also affect the evolution of good habits and aid in the prevention of spine problems [17, 20]. Teaching proper movement patterns and posture habits to elementary school children is very important to be able to integrate them into their daily behavior. Studies on the integration of back school programs into the educational context have shown that changing children’s back care knowledge, beliefs, habits, attitude, and abilities can significantly improve public health [1, 8, 21].

In terms of knowledge about the spine, in the Hungarian national core curriculum, the spine as a concept first appears in primary school, where they first learn about vertebrates and invertebrates, in general about bones and anatomy, but the concept of vertebrae or disc does not come up, they do not learn about muscles, muscular system, in addition, the correct posture is learned within the framework of physical education class, around one time a year, which they learn with gymnastics.

In contrast, the main topics of the child back school programs concerning the theory are: anatomy, biomechanics, ergonomics mainly focusing on the spine, and spine friendly lifestyle; concerning the practice are: trunk muscle strengthening, muscles stretching of the muscles responsible for posture, sensation and automatization of correct posture, and lumbar motor control ability improvement [8, 10, 14, 22, 23]. The content of the back school programs may be necessary to ensure children have sufficient back care knowledge for more effective prevention.

Questionnaires are suitable tools for examining back care knowledge. Before developing a questionnaire, it is essential to know what back care knowledge questionnaires exist for children and what age they are adapted to, is there any age group for which there is no adapted back care knowledge questionnaire. Concerning age groups, the content, wording, number and type of questions and answers should be examined when designing the questionnaire. There are two validated Spanish questionnaires on the topic of back care knowledge: the Health questionnaire about knowledge for health and back care related to the practice of physical activity and exercise for adolescents (HEBACAKNOW-PAE) for 13–18-year-olds [3], and the Health questionnaire on back care knowledge concerning physical activities in daily life (HEBACAKNOW) for adolescent for 14–17 years old children, whose version has not yet a cross-cultural adaptation in English, but it is available [1]. The Back-care Behavior Assessment Questionnaire (BABAQ) for schoolchildren is also a validated questionnaire in Iranian, measures the theory based, healthy spine-related back-care behavior including behavioral capability (skills, knowledge), self-efficacy, expectation beliefs and performance spine among fifth-grade girls [24]. The Iranian version is not available, and has no cross-cultural adaptation in English, however that version is available.

Child back school programs are recommended from the age of 4 [9, 25], during which back care knowledge is developed, thus it would be useful to examine children’s back care knowledge at an early age [16, 25]. There is no validated questionnaire in the literature examining the back care and spine disease prevention knowledge of children aged 6–10 years.

## Methods

### Aims

The study aimed to develop a questionnaire examining back care and spine disease prevention knowledge for children aged 6–10 years and testing its psychometric properties, which includes the main groups of the content of back school programs: anatomy, biomechanics, ergonomics, spine use habits, spine-friendly lifestyle, besides assessing the back care knowledge of children in this age group, among those who attended back school program or not.

### Study design

The cross-sectional quantitative study was conducted between 2016 and 2020 in Pécs, Hungary. The director of the schools provided a Declaration of Support. All the parents were informed about the process of the back school program and have provided written consent permitting their children to participate in the study. The data were processed anonymously and confidentiality based on the Data Protection Act of Hungary. The study was approved by the Institutional Review Board of the Regional Research Committee of the Clinical Center, Pécs, Hungary (No.: 8342-PTE 2020).

### Development procedure

Development of the Hungarian version of the questionnaire “*Gerinchasználattal és -prevencióval kapcsolatos tudást felmérő kérdőív 6-10 éves gyerekek számára (GEPT-6-10)”* (Additional files 1, 2) was based on using the validity criteria of the Delphi method [1, 3, 26], which phases were the following:

I. phase: Review of Hungarian and international literature. Collecting and selecting evidence indicators.

We performed a literature review according to the Preferred Reporting Items for Systematic Reviews and Meta-Analyses (PRISMA) statement [27] concerning the English and Hungarian literature, examining the back care knowledge by questionnaire among children. We excluded studies that examine pain, since our focus was on prevention. Records were identified through databases (PubMed, Scopus, Science Direct, Web of Science, Embase, Cochrane Library, MATARKA) and additional records were identified through other sources (Ph.D. thesis, congresses, etc.). Finally, we found three validated questionnaires [1, 3, 24] in the field of back care knowledge. For wider mapping, we also reviewed several sources related to disease-specific knowledge [17], postural habits [28], low back pain [29], back pain [30, 31], among adults and children, and studies in the field of back school, back/posture education [9, 10, 25, 27, 32]. We aimed to evaluate the knowledge that back care programs provide: anatomy, spine use habits, biomechanics, ergonomics and spine-friendly lifestyles.

II. phase: Development of the first version of the questionnaire, elaboration of questions, groups of questions (items). Evaluation of the first version by six independent experts.

The task of the six independent experts (a physiotherapist; a Ph.D. graduate physiotherapist; a Ph.D., assistant professor; and a doctor having experience and making research in the field of spine problems, low back pain; a pedagogue, and also a child psychologist) was to include the most relevant issues in the questionnaire connected to back care and spine disease prevention knowledge based on the scientific evidence found in the literature review. They assessed professionally, the content and linguistic adequacy of the questions, suitable for the age group, without causing difficulty in understanding, and considered how many questions children can be burdened with. They assessed in terms of content the level of difficulty of the questions and commented which response method would be the most appropriate.

III. phase: Development of the second version of the questionnaire. Evaluation of the second version by the target population (pilot testing).

After the expert’s opinions, the complied sets of questions were tested by a total of 15 children from different age groups. The most important, useful suggestions were registered. They were asked about the content, the ease, the form of the questionnaire, the number of questions, and lucidity of the language, and the interpretability of symbols given as answers.

IV. phase: Development of the final version of the questionnaire. Administration.

Comments from the target population were evaluated by the experts, and incorporated into previous professional decisions, thus making the final version. The structure and form of the presentation were also decided.

The English version “HEalth Questionnaire on BAck Care and Spine Disease Prevention Knowledge for 6-10 years old children (HEQBACK-6-10)” (Additional files 1, 2) of the questionnaire was translated by two experts, a synthesis was made from the two translated versions, and finally, a retranslation was carried out. Besides, the questionnaire was filled out by English bilingual voluntary children from the target population to make proposals for a better understanding of the questions in English. This version has not yet a cross-cultural adaptation.

### Study groups

A post-hoc sample size estimation (using G*power) for the correlation analysis (significance set at 5%, power set at 0.8, effects size at 0.15, and the number of predictors at 2) showed that the sample size was optimal, given the study power, i.e. 99.99% [33]. A total of 469 children were selected in the study by convenience sample selection. One of the participants left the school during the program, and five of them were excluded, because of missing data. Data of 463 children (220 boys, 243 girls) were processed in the study, their mean age was 7.51 ± 1.32 years. During the survey, we distinguished three groups according to age and grade, and three subgroups accordingly they took part in a back school program, e-learning back school program or none of them (Table 1). The protocol describing the back school programs and the study examining the effectiveness of the programs is under publication, during which we considered it important to test the back school program also in an online environment.

1. 230, 6–7 years old, 1. grader children (108 boys, 122 girls; mean age: 6.530 ± 0.500 years).
2. 119, 7–8 years old, 2. grader children (62 boys, 57 girls; mean age: 7.487 ± 0.502 years).
3. 114, 9–10 years old, 4. grader children (50 boys, 64 girls; mean age: 9.526 ± 0.502 years).

**Table 1:** Groups and subgroups in the examined population

Age, Class	Participated in BSP (persons)	Participated in e-learning BSP (persons)	Did not participate in BSP (persons)	Total (persons)
6–7 years, 1. grader	26	0	204	230
7–8 years, 2. grader	28	0	91	119
9–10 years, 4. grader	26	27	61	114
6–10 years, 1.- 4. grader	80	27	356	463

*BSP* back school program

#### Inclusion criteria

6–10 years old primary age children.

#### Exclusion criteria

Congenital or acquired spinal disease, severe locomotor, internal or neurological illness, non-mature children for school, children with special education needs (SEN), certified athletes, sports club members [9, 16, 22, 25].

### The applied back school programs

Both programs were led by two physiotherapists (Habil, P.hD., associate professor; MSc, P.hD. student), the back school program in person, the e-learning program online, the content and material of which was available on the website of https://gerincsuli.hu/ [34], has been developed by us, after an extensive literature review of back school, back education programs [9, 10, 15, 22, 25, 35, 36]. Each program took 12 weeks, one time per week, 45 min per occasion.

The theoretical part of the back school programs included anatomical knowledge: human skeleton, spinal column structure, function, bones of the spinal column, vertebrae, skeletal muscles, trunk muscles; biomechanical knowledge: spinal column movements (trunk flexion, extension, lateral flexion, rotation, elongation), structure of the movement segment, biomechanical properties of the movement segment and the disc; ergonomic knowledge; spine protection rules and elements of a spine-friendly lifestyle (correct posture in standing, sitting position, spine-friendly school, spine-friendly sports, spine-friendly leisure time) with devices and illustrations, in a playful form. The practical part included: trunk muscle strengthening, muscle stretching of the muscles responsible for the posture, sensation and automatization of correct posture, lumbar motor control ability development, and spine use, with and without tools, in a playful form [16, 34].

### Statistical analyses

The scores of the questionnaire were calculated, the mean and standard deviation values of the questions and categories were obtained. The normality of the continuous variables was tested by Kolmogorov Smirnow tests, *p*-value higher than 0.05 was considered a normally distributed score [37]. We used SPSS (*v.27*) software for Windows to make different statistical analyses.

### Validity and reliability testing of the GEPT-6-10

Internal consistency was tested by Cronbach’s alpha, which value could have been excellent (0.93–0.94), strong (0.91–0.93), reliable (0.84–0.90), robust (0.81), fairly high (0.76–0.95), high (0.73–0.95), good (0.71–0.91), relatively high (0.70–0.77), slightly low (0.68), reasonable (0.67–0.87), adequate (0.64–0.85), moderate (0.61–0.65), satisfactory (0.58–0.97), acceptable (0.45–0.98), sufficient (0.45–0.96), not satisfactory (0.4–0.55) and low (0.11) [38].

Test-retest reliability was tested by ICC (intraclass correlation coefficients, using 95% of confidence interval) in 463 participants [39].. The ICC values can range from 0 and 1 and, the values of less than 0.5, between 0.5 and 0.75, between 0.75 and 0.9, and greater than 0.90 are indicative of poor, moderate, good, and excellent reliability, respectively [40]. The form of data collection was the same in the back school programs and non-back school program groups. All the children from the non-back school group filled the questionnaire twice with an interval of 7 days. As well, all the children from the back school program groups filled the questionnaire twice, first at the end of the back school program, then 7 days later.

The standard error of measurement (SEM = standard deviation of all scores × square root of (1 − ICC) and 95% of minimal detectable change were calculated to multiplying SEM by 2,77) estimates the absolute reliability [39].

The mean difference between the two measurement intervals and the 95% limits of agreement (LoA) was calculated by LoA = mean difference (d) ± 1.96 SD of the mean differences. The Bland–Altman (BA) plot was used to visually examine the 95% limits of agreement between the test and retest total scores, where narrower LoAs suggested better agreement at the individual level [41, 42]. This association was examined by linear regression analysis [1]. The convergent validity was tested by Spearman’s rank correlation coefficients [43]. The discriminant validity pertains to the ability of a measurement system to determine differences between two groups that are diverse differently from each other concerning the parameter that is tested [44]. In the study, the discriminant validity was tested to compare the results of the questionnaire’s scores between the non-back school and back school groups of different ages to examining the difference between them.

## Results

### Validity and reliability

#### Content validity questionnaire

Development of the questionnaire started with the selection of topics related to back care and spine disease prevention knowledge included in back school programs, specified by several back school, back education programs and questionnaires examining back care knowledge. Table 2 shows the validated questionnaires of back care knowledge for children, found in the Hungarian and English literature.

**Table 2:** The list of validated back care knowledge questionnaires until the age of 18 years old, found in the Hungarian and English literature

Author (year)	Examined population	Questionnaire
Miñana-Signes V et el. (2015) [3]	• 230 students • 13–18 years	Conocimientos sobre la Salud y Cuidados de la Espalda relacionados con la Actividad y Ejercicio físico (COSACUES-AEF) Health questionnaire on back care knowledge concerning practice physical activity and exercise for adolescents (HEBACAKNOW-PAE)
M. Monfort et al. (2016) [1]	• 171 students • 14–17 years	Health questionnaire on back care knowledge in daily life physical activities (HEBACAKNOW)
Akbari-Chehrehbargh Z et al. (2020) [24]	• 610 students • 5th grade	Back-care Behavior Assessment Questionnaire (BABAQ)

Main topics included in a back school program: anatomy, biomechanics, ergonomics mainly focusing on the spine, spine use, and spine friendly lifestyle. Formulation of the items started accordingly, and ten preliminary items were prepared for the questionnaire. According to the suggestions of the experts, we minimalized the numbers of the questions for this age group, not to overload them, and we highlighted the most essential issues, for this reason, seven questions were left. Linguistically, the first wording of the seven questions has been transformed, which developed as follows: Question 1 “Draw the spines in the pictures!” “Draw all the spinal columns in the pictures!”, Question 2 “Completely color all the vertebrae blue and all the discs red!” “Color one vertebra to blue and one disc to red!”, Question 3 “What are the correct postures while watching TV? More answers are possible!” “Mark 2 correct postures during watching TV!”, Question 4 “Circle the correct postures! More answers are possible!” “Mark 3 correct postures!”, Question 5 “Connect those with similar hardness!”, Question 6 “Circle where the boy lifts the bag correctly!” “Mark where the boy is correctly lifting the bag!” and Question 7, “What holds and moves the spine?” “Mark what holds and moves the spinal column?”. As the questionnaire can be filled by children who cannot read or write, we have provided pictures and symbols at most of the questions for choosing the answer. After the changes, the assessment of 15 children followed. In their opinion, the last question where children had to figure out for themselves what holds and moves the spine, instead, it would be better if they could choose the correct answer from two drawn symbols. They also confirmed that the questions were understandable. An adult read aloud the questions, that already included the instructions, highlighting what to do, how to answer, if more than one answer were correct, it was given how many. The accepted final version included a total of 7 questions, of which question 1, 2, 5, 7 goes under the category of “anatomy and biomechanics (category 1)”, and question 3, 4, 6 are in the category of “spine use, ergonomics and spine friendly lifestyle (category 2)”. There are questions, with more correct answers, for every correct answer a point can be given, thus who can find all the correct answers a total of 7 points can be given for question 1, 2 points for question 2, 2 points for question 3, 3 points for question 4, 2 points for question 5, 1 point for question 6, and 1 point for question 7. For the wrong answer, 0 point was given. A maximum of 18 points can be obtained in the questionnaire and a minimum of 0 point. The criteria for the correct answers to each question are provided in Table 3.

**Table 3:** Criteria and correct answers

Question	Criteria and correct answer
1	The spinal columns have to be drawn from head to pelvis and also the shape of the spinal columns have to be drawn correctly.
2	One vertebra has to be colored to blue, and one disc to red.
3	^a^Number 4 and 5 are correct.
4	^a^Number 2, 3, and 4 are correct.
5	One vertebra has to be connected to the Lego, and one disc to the ball.
6	^a^The boy is correctly lifting the bag on the first drawing.
7	^a^The muscles hold and move the spinal column, the second drawing shows a muscle.

^a^The numbering of the images in the questionnaire should be considered line by line from left to right for each question, starting with the number 1

#### Internal consistency

The internal consistency of the questionnaire was determined using Cronbach’s alpha values. For the total 7 items, Cronbach’s alpha was α = 0.797 (0.768–0.824), the questions correlated well with each other, confirming our hypothesis. The pairs of each question, category, and total scores correlated significantly (*p* < 0.001). The results corroborated, that the questionnaire showed good internal consistency.

#### Test-retest reliability

The reliability of the questionnaire was also examined using the test-retest method by intraclass correlation coefficient (ICC). The correlation coefficient was strong (0.989) for the total scores, and ranged from moderate to strong (0.742–0.975) for the questions (*p* < 0.001), with minimal SEM and MDC95 (0.606 and 1.680 respectively) (Table 4).

**Table 4:** Test-retest reliability of the Health Questionnaire on Back Care and Spine Disease Prevention Knowledge for 6–10 years old children

	**Mean test** **(SD)** **(point)**	**Mean retest** **(SD)** **(point)**	**Difference** **between** **test, retest** **(SD)** **(point)**	ICC	CI 95%		p	**SEM** **(point)**	**MDC95** **(point)**
					lower	upper			
**1**	2.063 (2.639)	2.413 (2.558)	−0.350 (0.808)	0.975	0.970	0.979	p < 0.001	0.411	1.139
**2**	0.851 (0.950)	0.952 (0.910)	−0.102 (0.456)	0.936	0.923	0.947	p < 0.001	0.235	0.652
**3**	0.706 (0.830)	0.877 (0.830)	−0.171 (0.482)	0.908	0.889	0.923	p < 0.001	0.252	0.698
**4**	1.240 (1.214)	1.382 (1.182)	−0.143 (0.544)	0.946	0.935	0.955	p < 0.001	0.278	0.771
**5**	0.849 (0.951)	0.937 (0.906)	−0.089 (0.459)	0.935	0.922	0.946	p < 0.001	0.237	0.656
**6**	0.788 (0.409)	0.801 (0.399)	−0.013 (0.254)	0.890	0.868	0.908	p < 0.001	0.134	0.372
**7**	0.330 (0.471)	0.564 (0.496)	−0.233 (0.438)	0.742	0.690	0.785	p < 0.001	0.246	0.681
**Total**	6.827 (5.979)	7.927 (5.577)	−1.099 (1.218)	0.989	0.987	0.991	p < 0.001	0.606	1.680

*CI* confidence interval, *ICC* intraclass correlation coefficient, *SEM* standard error of measurement, *MDC95* minimal detectable change at 95%

**Fig. 1:**
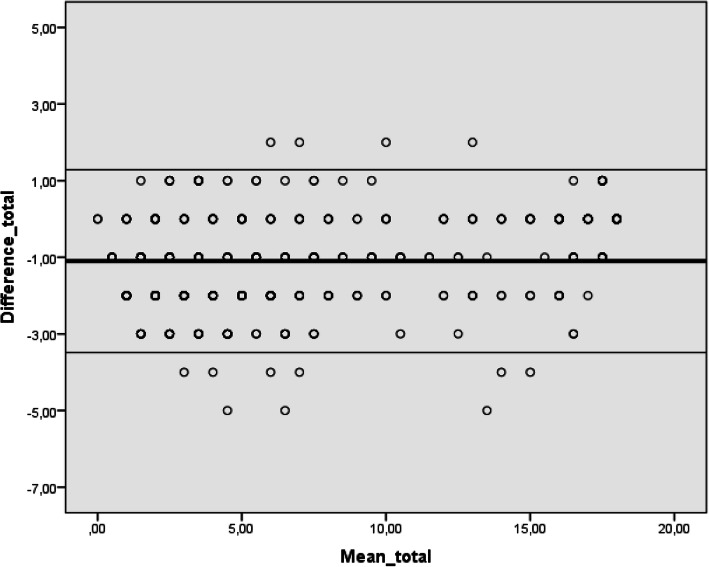
Bland–Altman plot of the differences between test 1 and 2 (retest) for the total score of the Health Questionnaire on Back Care and Spine Disease Prevention for 6–10 Years Old Children. The observed mean of agreement (solid lines) and limits of agreement (dashed lines) are presented within ±2 standard deviations (*n = 463*). x-axis: Mean of the total scores of the Health Questionnaire on Back Care and Spine Disease Prevention Knowledge for 6–10 years old children between test 1 and 2; y-axis: Differences of the total scores of the Health Questionnaire on Back Care and Spine Disease Prevention Knowledge for 6–10 years old children between test 1 and 2. ______observed average agreement, __________95% limit of agreement

The Bland–Altman plot and the limits of agreement concerning the total score of the questionnaire (− 1.10; − 3.49-1.29–0.30 points) are shown in Fig. 1. The test–retest differences of the total score increased as the acquired sum of score increased (F = 56.89, *p* < 0.001, Constant: 9,10, Beta coefficient = 1.56; *p <* 0.001).

The value of mean difference was − 1.10 (SD ± 1.22), and the limits of agreement for the total HEQBACK scores were − 3.49 and 1.29 points (Fig. 1).

#### Convergent validity

Convergent validity was examined using Spearman’s rank correlation analysis between total score and age, where we found a weak but significant association (*R =* 0.171, *p <* 0.001) [43].

#### Discriminant validity

Discriminant validity was tested among children who took part in a back school program or not among different age groups. The Kolmogorov-Smirnov test results showed non normally distributed scores of the questionnaire (*p* > 0.05). We found significant differences in the back care knowledge between 6 and 7 years old (*p <* 0.001), 7–8 years old, and also 9–10 years old groups. Table 3 summarizes the results of back care knowledge in the examined population. The highest total score was 17.115 ± 0.909 points among 9–10 years old children in the back school program group. The second highest total score was 16.308 ± 2.429 points among 6–7 years old children, who took part in a back school program. E-learning back school program seemed to be similarly effective according to the total scores among 9–10 years old children (15.926 ± 3.037 points), than the back school program for 7–8 years old children (15.714 ± 1.802 points) (Table 5).

**Table 5:** The results of the back care and spine disease prevention knowledge in the examined population

		6–7 years, 1.grader		7–8 years, 2.grader		9–10 years, 4.grader			**6–10 years,** **1–4.grader**
		No participation in BSP (***n*** = 204)	Participation in BSP (***n*** = 26)	No participation in BSP (***n*** = 91)	Participation in BSP (***n*** = 28)	No participation in BSP (***n*** = 61)	Participation in BSP (***n =*** 26)	Participation in e-learning BSP (***n*** = 27)	**Total of participants** ***(n = 463)***
**Q1** **(point)**	**Mean**	1.088	6.231	0.319	5.643	0.705	6.808	6.074	2.063
	**SD**	1.623	1.142	0.880	1.367	0.803	0.492	1.662	2.639
**Q2** **(point)**	**Mean**	0.691	1.923	0.187	2.000	0.525	1.962	1.741	0.851
	**SD**	0.946	0.392	0.469	0.000	0.721	0.196	0.526	0.950
**Q3** **(point)**	**Mean**	0.505	1.615	0.176	1.536	0.443	1.885	1.741	0.706
	**SD**	0.691	0.637	0.437	0.637	0.671	0.326	0.594	0.830
**Q4** **(point)**	**Mean**	1.054	2.654	0.286	2.679	0.721	2.885	2.593	1.240
	**SD**	1.037	0.977	0.583	0.476	0.897	0.326	0.747	1.214
**Q5** **(point)**	**Mean**	0.637	1.923	0.341	1.929	0.557	1.654	1.889	0.849
	**SD**	0.902	0.392	0.619	0.378	0.904	0.629	0.423	0.951
**Q6** **(point)**	**Mean**	0.765	1.000	0.681	1.000	0.689	0.962	0.963	0.788
	**SD**	0.425	0.000	0.469	0.000	0.467	0.196	0.193	0.409
**Q7** **(point)**	**Mean**	0.118	0.962	0.099	0.929	0.311	0.962	0.926	0.330
	**SD**	0.323	0.196	0.300	0.262	0.467	0.196	0.267	0.471
**C1** **(point)**	**Mean**	2.534	11.038	0.945	10.500	2.098	11.385	10.630	4.093
	**SD**	2.432	1.280	1.508	1.427	1.630	0.898	2.041	4.256
**C2** **(point)**	**Mean**	2.324	5.269	1.143	5.214	1.852	5.731	5.296	2.734
	**SD**	1.608	1.343	0.973	0.876	1.389	0.452	1.354	2.031
**Total score (point)**	**Mean**	4.858	16.308	2.088	15.714	3.951	17.115	15.926	6.827
	**SD**	3.500	2.429	2.053	1.802	2.156	0.909	3.037	5.979
**p**		^●^p < 0.001		^●^p < 0.001		^●●^p < 0.001			

^●^Mann-Whitney test results, ^●●^Cruscal-Wallis test results, *BSP* back school program, *Q* question, *C1* category 1; C2: category 2; *SD* standard deviation

## Discussion

The most important results of the study show that we have developed a valid and reliable questionnaire for assessing the back care and spine disease prevention knowledge for 6–10 years old children. The instrument was validated on Hungarian population, but an English version is also available. The validation procedure was according to the Delphi method, involving experts and children from the target population, thus helping to make interpretable and professionally relevant questions [1, 28].

According to the teachers and children opinion, filling the questionnaire was neither too easy nor too difficult, thanks to the given answer options, which were pictures and symbols, that also makes it easier to fill out.

Psychometric properties support the reliability of the instrument. The validity and reliability results showed good stability of the total score (Cronbach 0.797). The test-retest reliability results showed a strong correlation, the ICC was strong in total scores and in case of all questions. The limit of agreement was relatively low and suggested a narrow error of measurement range (− 3.49–1.29) and the mean difference between the two measurements was − 1.10, which result showed a low systematic error and small difference between the test and retest measurements. Furthermore, the regression analysis showed that the differences of the total score values increased as the acquired scores increased (*p* < 0.001). For those who reached a higher score on the first measurement, the results of the second measurement showed an even greater improvement, they got to know the questions when completing the questionnaire and were better suited to correct them. The HEQBACK total scores showed a significant correlation with age (*p <* 0.001) and in every age group the difference was significant between the subgroups which proved the higher scores of back school groups (*p <* 0.001).

Validated questionnaires existing in the literature measuring back care knowledge in other age groups. Miñana-Signes et al. validated the Health questionnaire about knowledge for health and back care related to the practice of physical activity and exercise for adolescents (HEBACAKNOW-PAE) for 13–18 years old, [3], M. Monfort et al. validated the Health questionnaire on back care knowledge in daily life physical activities (HEBACAKNOW) for children aged between 14 and 17 years old [1]. Akbari-Chehrehbargh et al. developed the Back-care Behavior Assessment Questionnaire (BABAQ) for schoolchildren (5th grade), which aimed to measure the theory-based content of back care programs [24]. It is worth mentioning a validated questionnaire connected to postural habits, validated by M. Monfort and Miñana-Signes in 2020, the questionnaire of Back-health related postural habits in daily activities (BEHALVES) for 13–17-year old adolescents, that occupies in some terms with back care knowledge [28].

However, Health Questionnaire on Back Care and Spine Disease Prevention Knowledge for 6–10 years old children is the first questionnaire validated by professionals for children at that early age to assess the back care and spine disease prevention knowledge. The study population consisted of children who took part in back school program or not. The validity and reliability of the questionnaire were good, it is a suitable instrument for the assessment of back care knowledge of 6–10 years old children.

It is interesting to look at how low the back care knowledge of children not participating in any back school or posture education program. In the study of Miñana-Signes V et al., 5th-grade primary school children who had not yet received back educational program (control group, mean age: 11.13 ± 0.34 years) completed two validated questionnaires related to back care knowledge. At HEBACAKNOW-PAE 2.04 ± 0.90 points were obtained from the maximum 10 points (20.4%), at HEBACAKNOW-DL 2.43 ± 1.18 points were achieved out of the maximum 10 points (24.3%) [15]. In the recent study children who did not participate in BSP reached 4.86 ± 3.500 points (1st grader) (27.0%), 2.09 ± 2.05 points (2nd grader) (11.6%), and 3.951 ± 2.16 points (4th grader) (22.0%) compared to the maximum 18 points. If we look at the percentage of correct answers, it can be deduced that children’s knowledge of back care, spinal use and prevention is between 20 and 60%, most are closer to 20%, which is inadequate.

It is also interesting to observe the back care knowledge of children after a back school program. Table 6 shows the examined population, and the results of back care knowledge assessed by validated and not validated knowledge questionnaires, tests, and the results of the recent study.

**Table 6:** Comparison of the results of back care knowledge in the intervention groups

Author (year)	Examined population/ Intervention group	Questionnaire/ Test	Total scores of back care knowledge for the intervention group (point)	
			Pre-intervention	Post-intervention
Miñana-S et al. (2019) [15]	• 11.19 ± 0.4 years • 16 students • 7 sessions (1 theoretical, 6 practical) of education	HEBACAKNOW-PAE (validated) HEBACAKNOW-DL (validated)	2.36 ± 0.72 3.32 ± 1.24	6.56 ± 1.28 6.32 ± 1.57
Dullien et al. (2018) [45]	• 10.59 ± 0.438 years • 87 pupils • 10-month education	Knowledge test (not validated)	14.42 ± 3.03	17.17 ± 2.84
Natália et al. (2017) [46]	• 8.8 ± 1.1 years • 44 children • 8-week education	Questionnaire to evaluate the theoretical knowledge of the spine and body posture (not validated)	–	9.0 ± 1.8
Rahele et al. (2012) [47]	• 203 students • 10–11 years • 4 educational pamphlets	Questionnaire of knowledge and behavior (locally validated)	Knowledge: 43.4 ± 12.93 Behavior: 53.3 ± 16.34	Knowledge: 60.5 ± 24.32 Behavior: 65.5 ± 20.34
Fabiana et al. (2011) [48]	• 9–16 years • 4th to 8th grade • 392 students at the baseline • 2 lessons and 1 practical lesson for education	Back care questionnaire (not validated)	3.6 ± 2.9	7.5 ± 2.2
Greet C et al. (2000) [32]	• 10.02 years • 82 children • 6-week education	Knowledge test (not validated)	−0.9	3.38
The recent study	• 6–7 years • 26 children • back school program	Health Questionnaire on Back Care and Spine Disease Prevention Knowledge for 6–10 years old children	–	16.308 ± 2.429
The recent study	• 7–8 years • 28 children • back school program	Health Questionnaire on Back Care and Spine Disease Prevention Knowledge for 6–10 years old children	–	15.714 ± 1.802
The recent study	• 9–10 years • 26 children • back school program	Health Questionnaire on Back Care and Spine Disease Prevention Knowledge for 6–10 years old children	–	17.115 ± 0.909
The recent study	• 9–10 years • 27 children • e-learning back school program	Health Questionnaire on Back Care and Spine Disease Prevention Knowledge for 6–10 years old children	–	15.926 ± 3.037

The back care and spine disease prevention knowledge need to be developed in addition to posture habits for the improvement of more effective spine prevention.

It may be useful to monitor how knowledge changes after a back school program, and to examine how children’s knowledge lasting in long term or maybe an update is required, if yes, when, besides to examine is there any direct impact on the prevention of the spine problems.

Another research is under publication that examines the effectiveness of back school programs in terms of back care knowledge, and compares the results of different kinds of back school programs.

In addition, it would be interesting to detect the back care knowledge of preschool children and to assess the effect of an educational program on the development of knowledge, since the material covers the back care knowledge of children 4–10 years.

## Conclusions

According to the results of the recent study, we can state that the questionnaire proved to be a valid and reliable tool for the examination of back care and spine disease prevention knowledge of 6–10 years old children. Back school programs have a remarkable impact on back care knowledge, the level of knowledge increases with the development of back schools.

### Limitations

The study was not randomized. A larger number of the examined population would have led to more certain conclusions. The instrument does not collect questions on physical exercise for back care. It would be useful to further adapt the questionnaire even more to age and to expand it with questions.

## Supplementary Information

**Additional file 1.** Gerinchasználattal és -prevencióval kapcsolatos tudást felmérő kérdőív 6–10 éves gyerekek számára.

**Additional file 2.** Health Questionnaire on Back Care Knowledge and Spine Disease Prevention for 6–10 Years Old Children.

## Data Availability

The datasets generated and analyzed during the current study are not publicly available due to concern for the parents of participants but are available from the corresponding author on reasonable request.

## Abbreviations

LBP: Low back pain
SEN: Special education needs
GEPT-6-10: Gerinchasználattal és -prevencióval kapcsolatos tudást felmérő kérdőív 6–10 éves gyerekek számára
HEQBACK-6-10: Health Questionnaire on Back Care and Spine Disease Prevention Knowledge for 6–10 years old children
BSP: Back school program
