# Supplementary material for: Health questionnaire on back care knowledge and spine disease prevention for 6–10 years old children: development and psychometric evaluation
Source: BMC Musculoskelet Disord. 2021 Sep 23;22:820. doi: 10.1186/s12891-021-04667-x (PMC8461832; doi:10.1186/s12891-021-04667-x)

**Additional file 1**

***Gerinchasználattal és -prevencióval kapcsolatos tudást felmérő kérdőív
6-10 éves gyerekek számára***

Név:

Nem:

Életkor:

Osztály:

Dátum:

1. Az összes gerincoszlopot rajzold be a képeken!

| 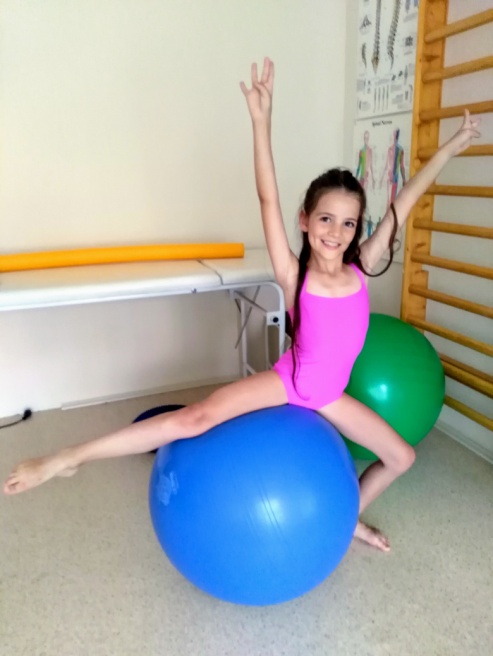 | 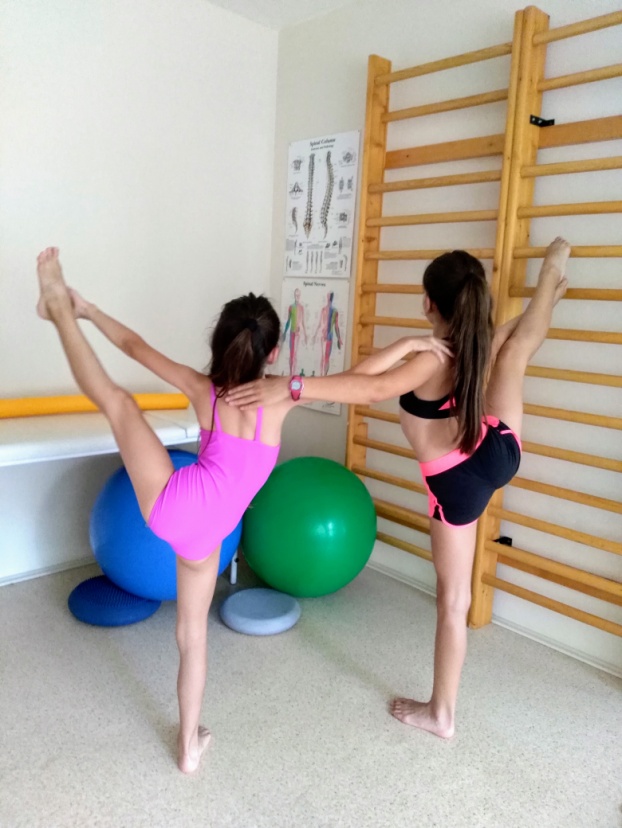 |
| --- | --- |
| 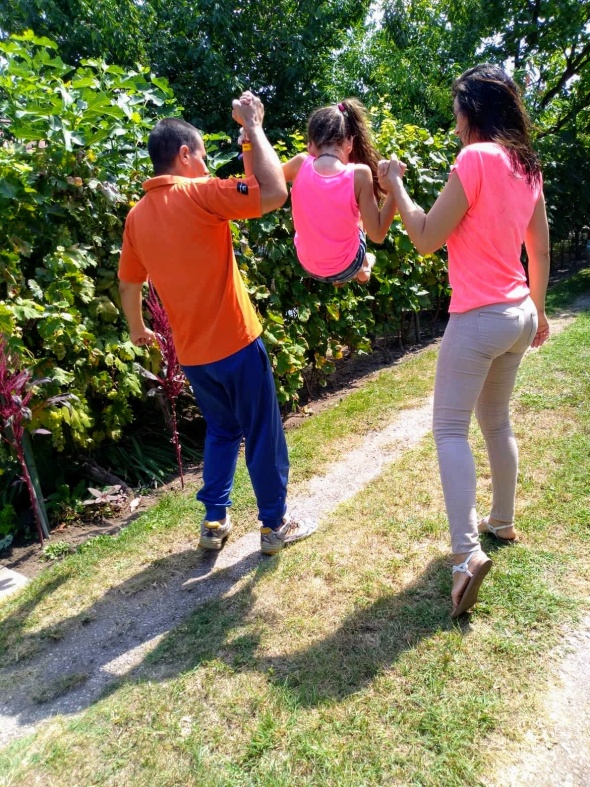 | 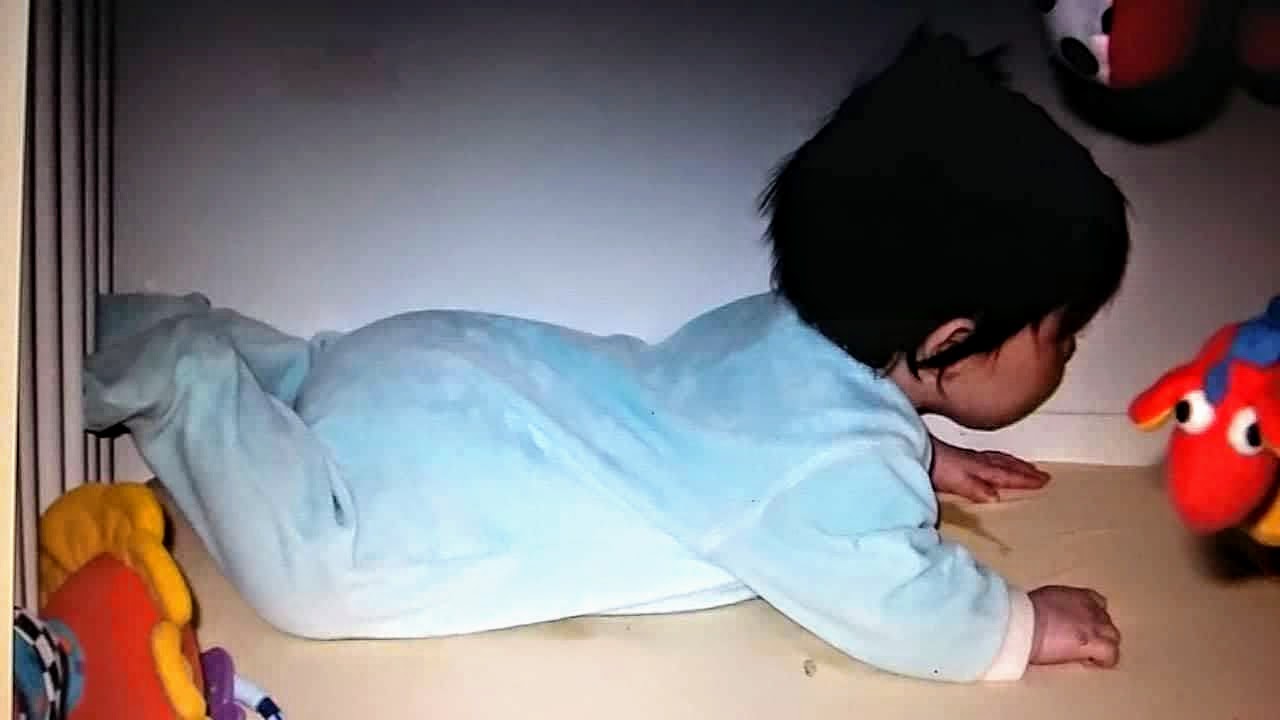 |
|  | |

1. Színezz ki egy csigolyát kékre, és egy porckorongot pirosra!


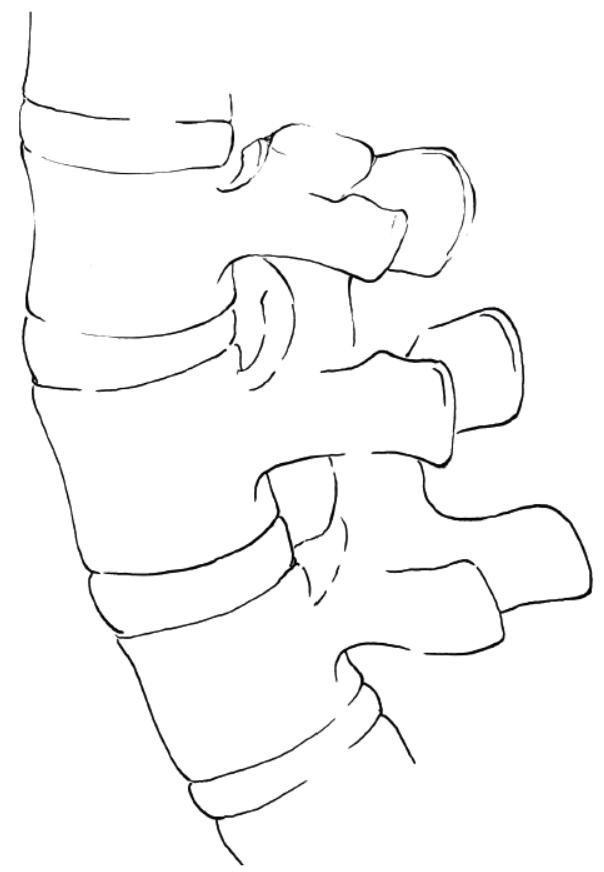


1. Jelölj be 2 helyes testhelyzetet TV nézés közben!

| 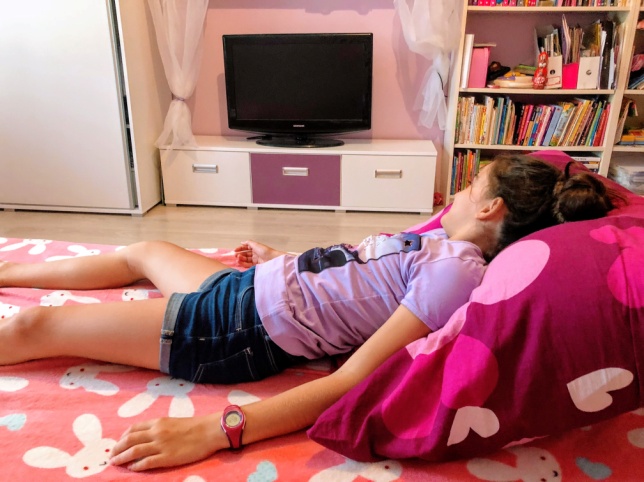 | 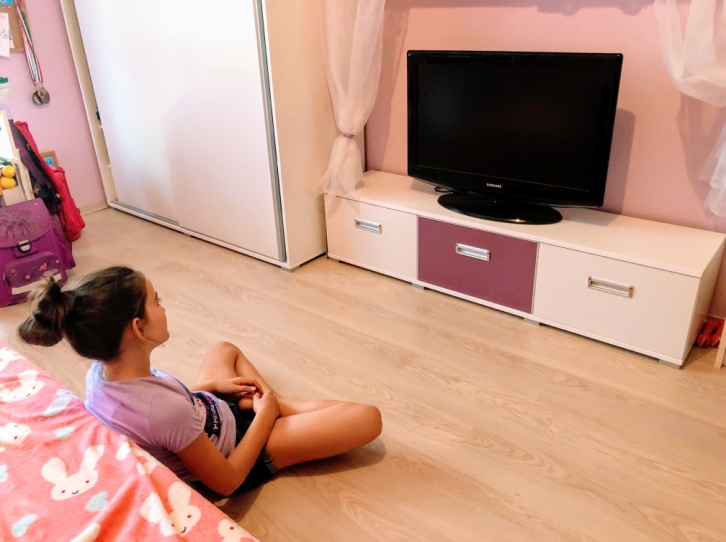 |
| --- | --- |
| 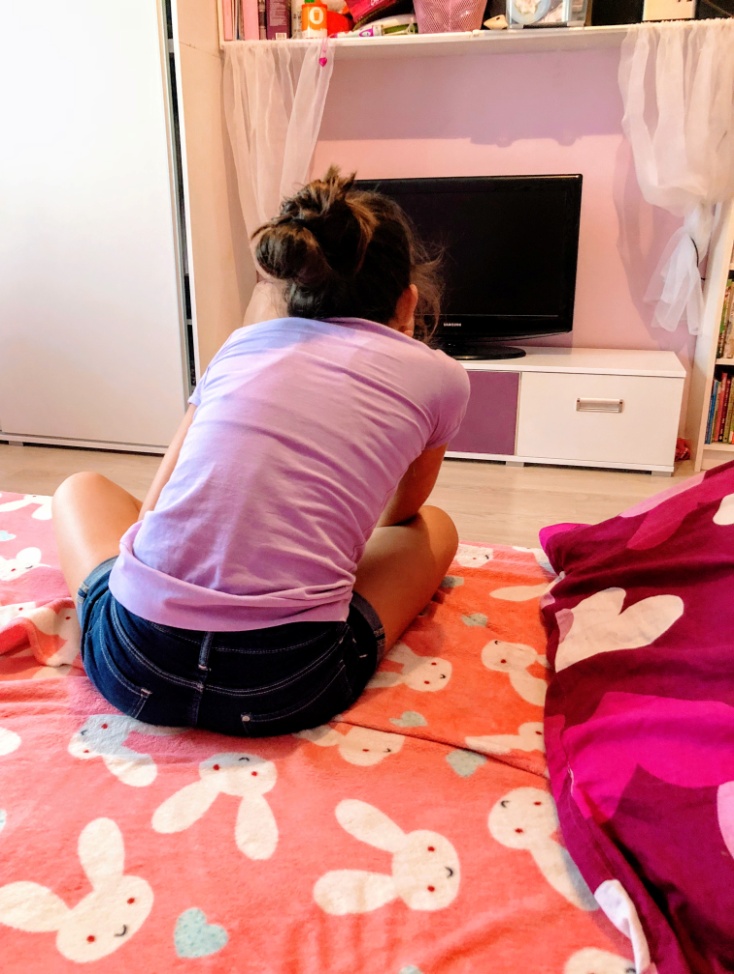 | 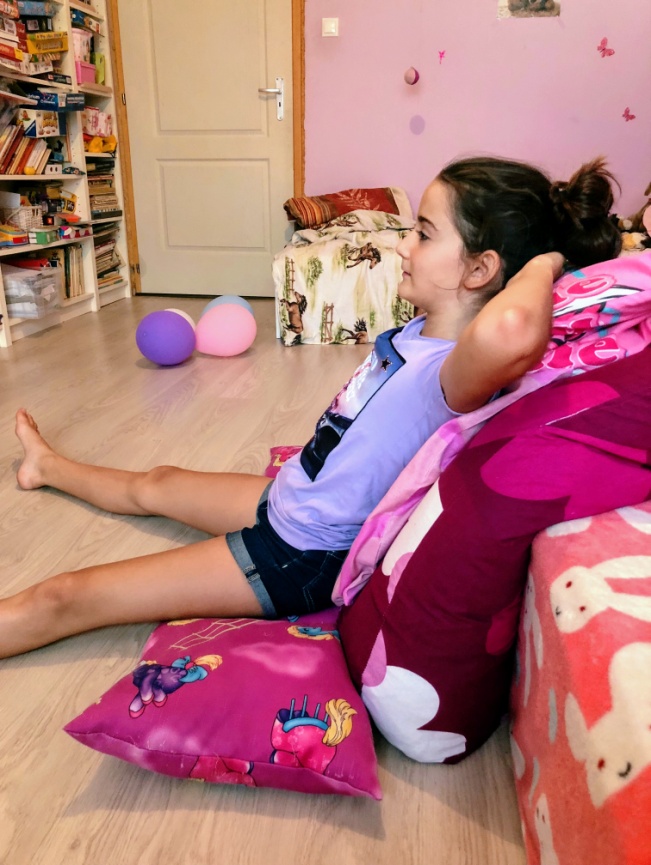 |
| 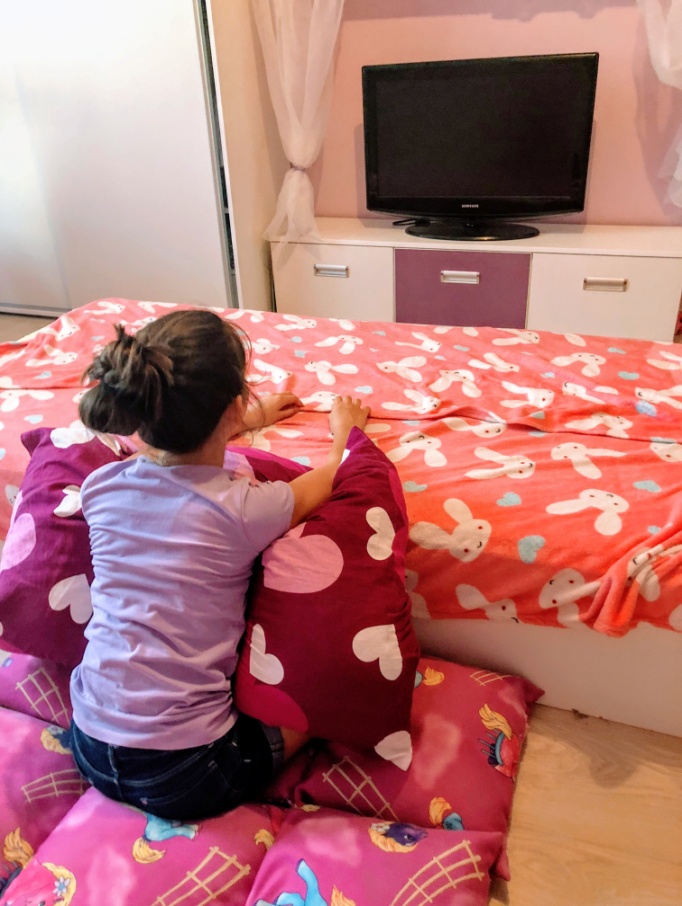 | |
|  | |

1. Jelölj be 3 helyes testhelyzetet!

| 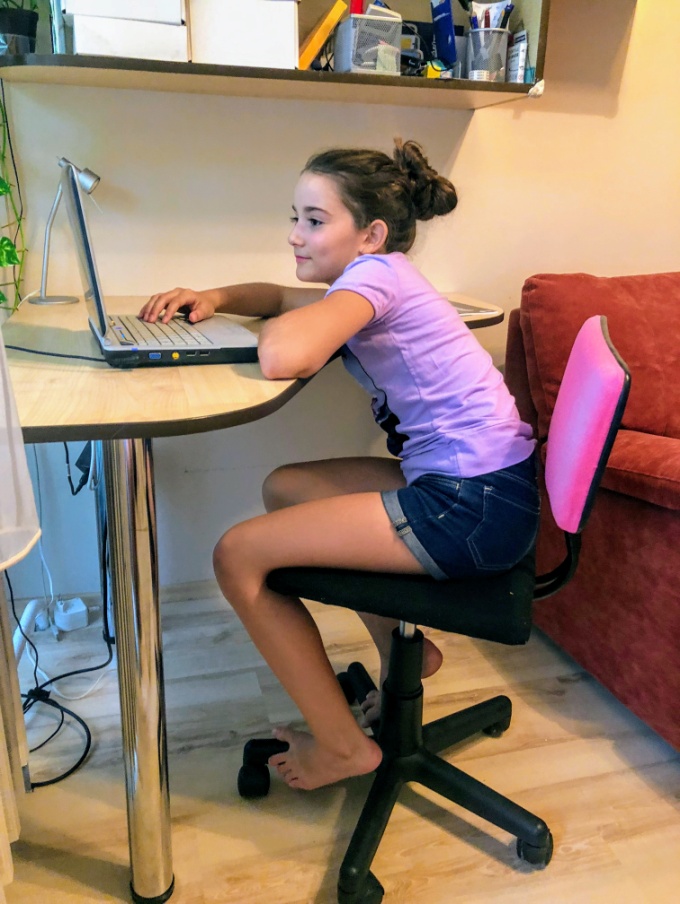 | 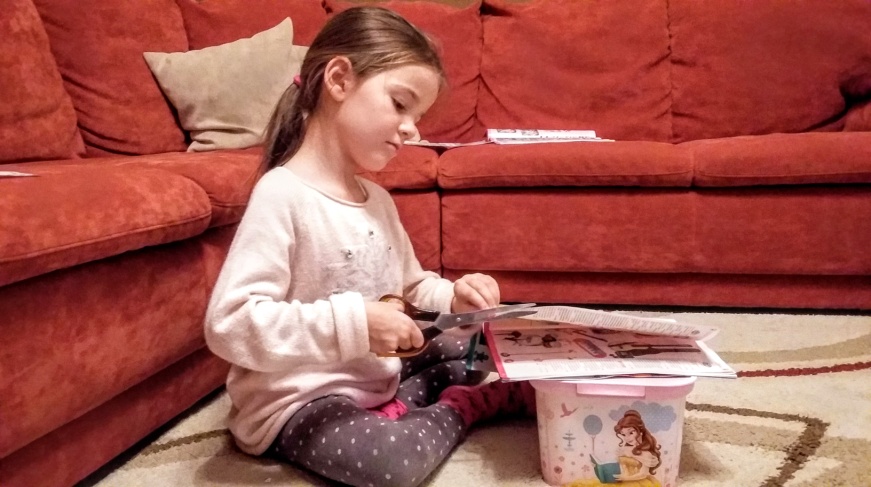 |
| --- | --- |
| 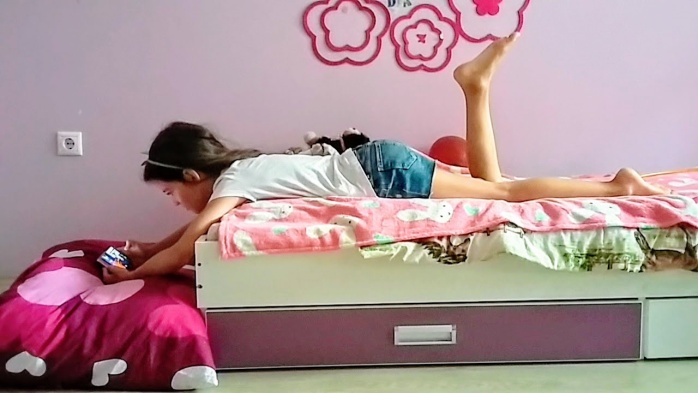 | 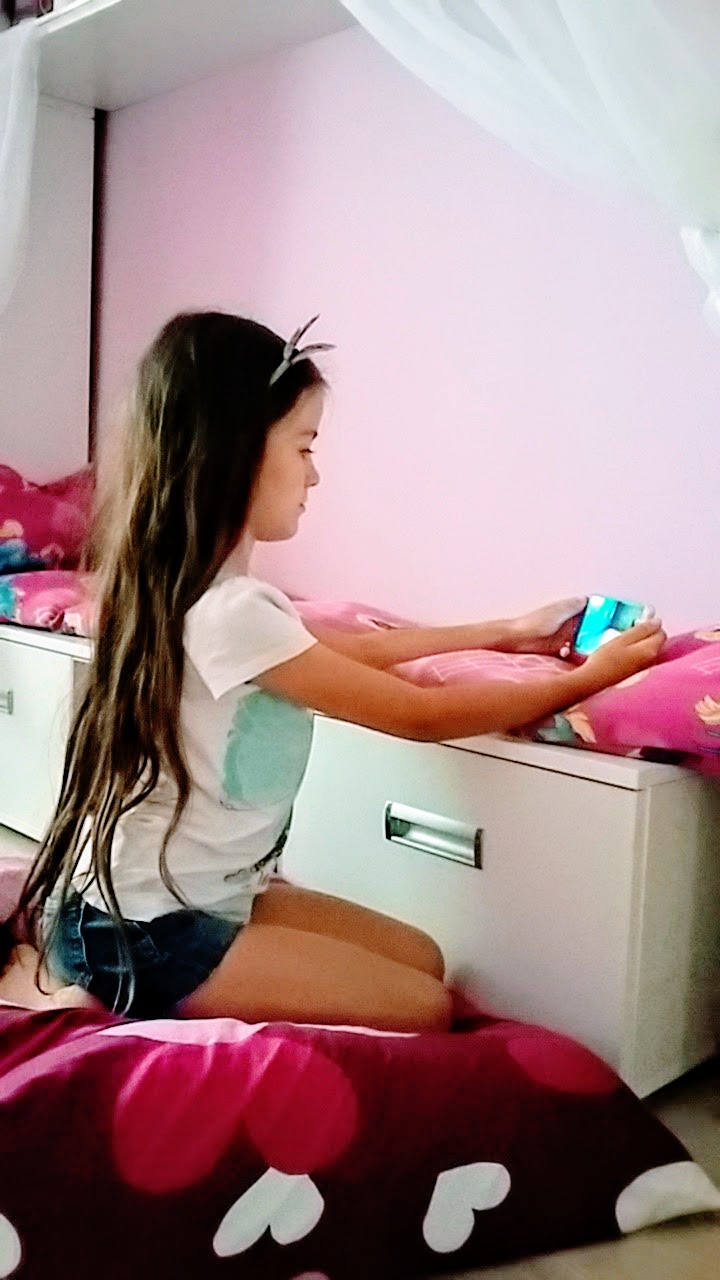 |
| 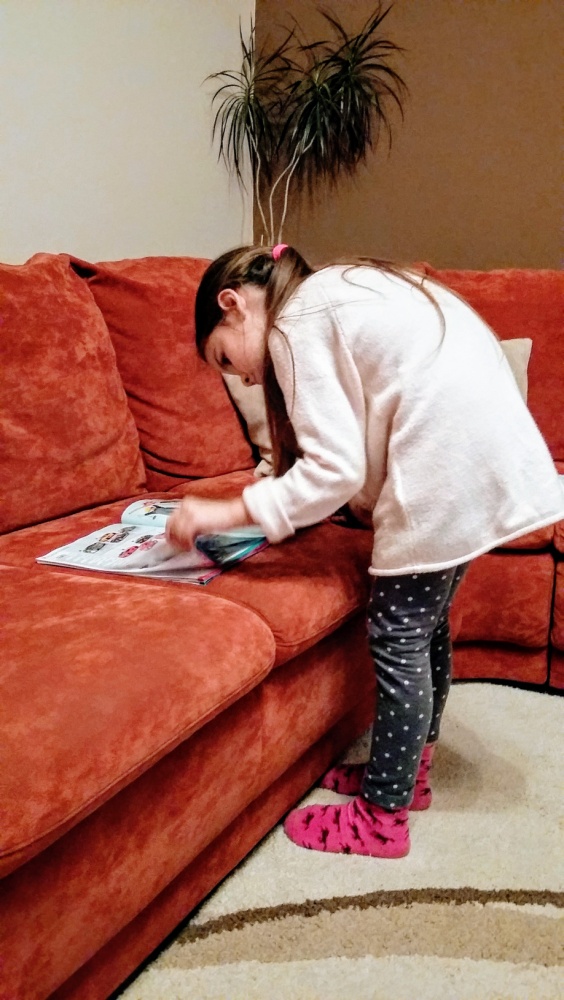 | 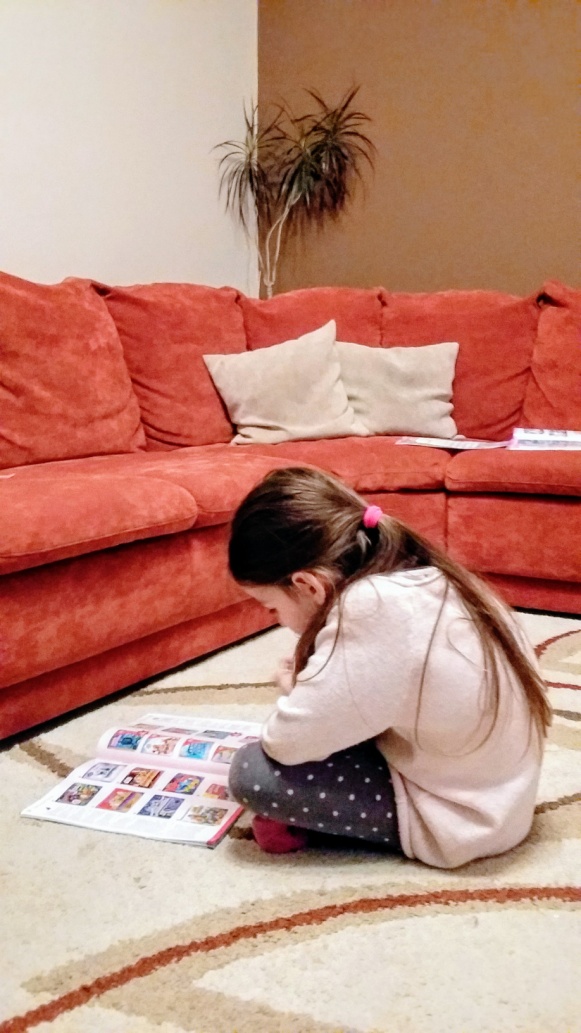 |
|  | |

1. Kösd össze a hasonló keménységűeket!

| 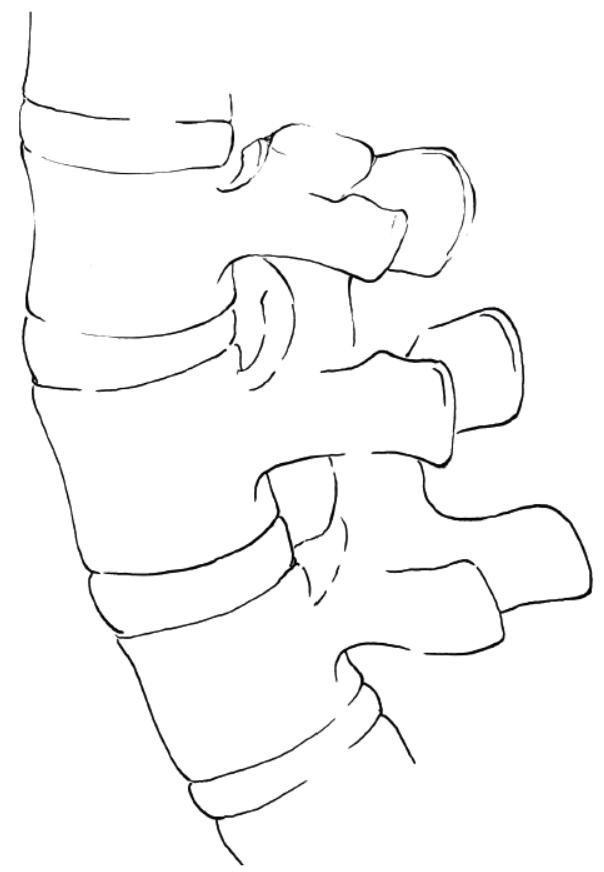 | 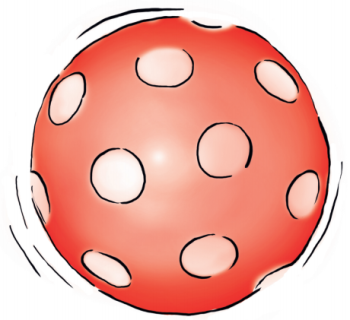 |
| --- | --- |
|  | 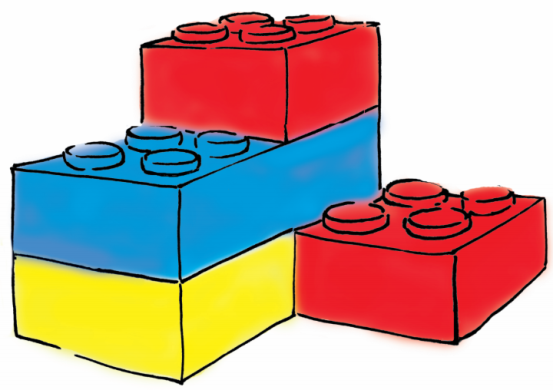 |
|  |  |

1. Jelöld be, hol emeli fel helyesen a fiú a táskát!


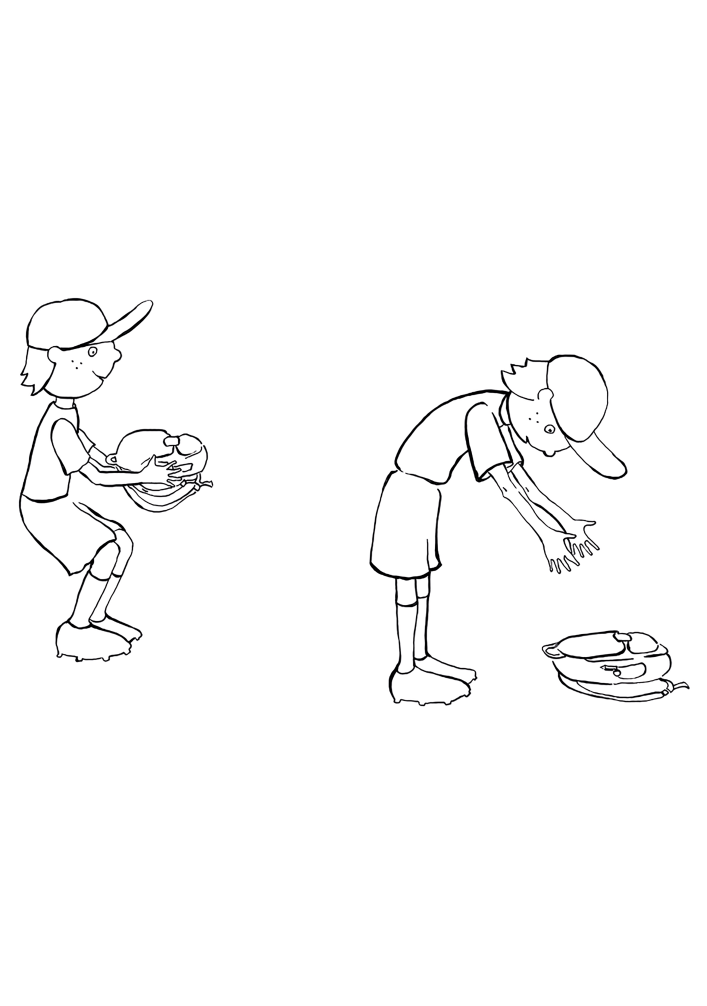


1. Jelöld be, mi tartja és mozgatja a gerincoszlopot?


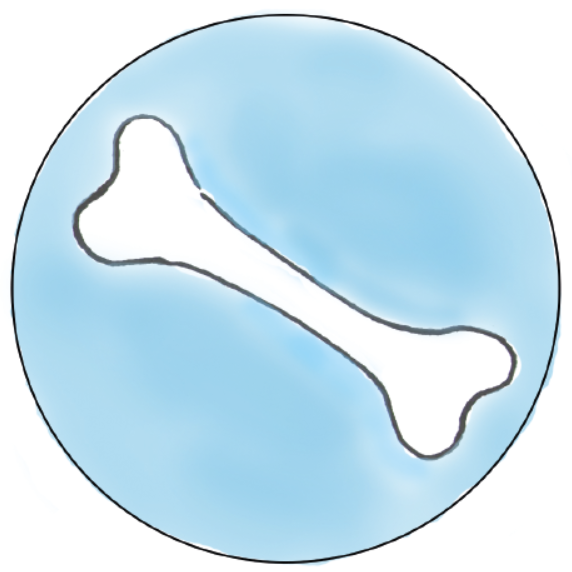

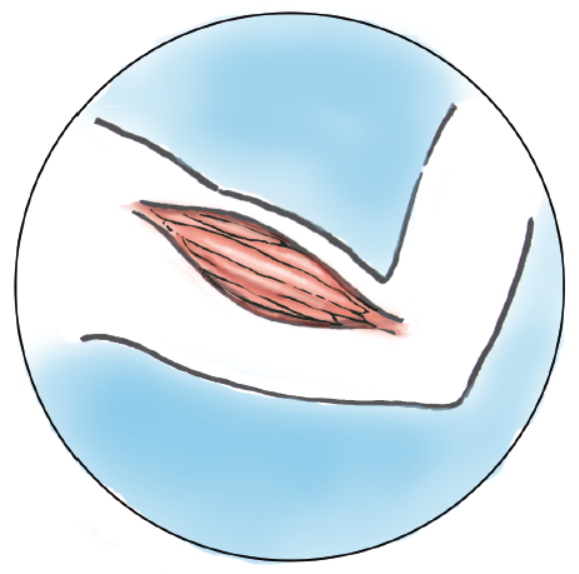

Supplement: Supplementary file 1 — Additional file 1. Gerinchasználattal és -prevencióval kapcsolatos tudást felmérő kérdőív 6–10 éves gyerekek számára. [file 12891_2021_4667_MOESM1_ESM.docx]
