# Supplementary material for: Health questionnaire on back care knowledge and spine disease prevention for 6–10 years old children: development and psychometric evaluation
Source: BMC Musculoskelet Disord. 2021 Sep 23;22:820. doi: 10.1186/s12891-021-04667-x (PMC8461832; doi:10.1186/s12891-021-04667-x)

**Additional file 2**

**Health Questionnaire on Back Care Knowledge and Spine Disease Prevention for 6-10 Years Old Children**

Name:

Sex:

Age:

School, class:

Date:

1. Draw all the spinal columns on the pictures!

| 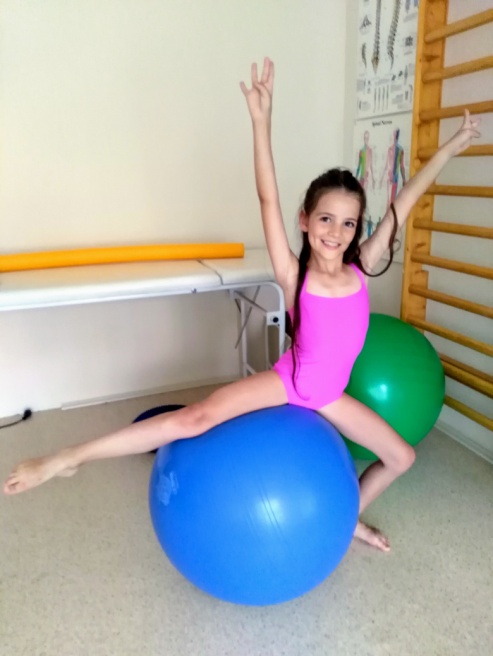 | 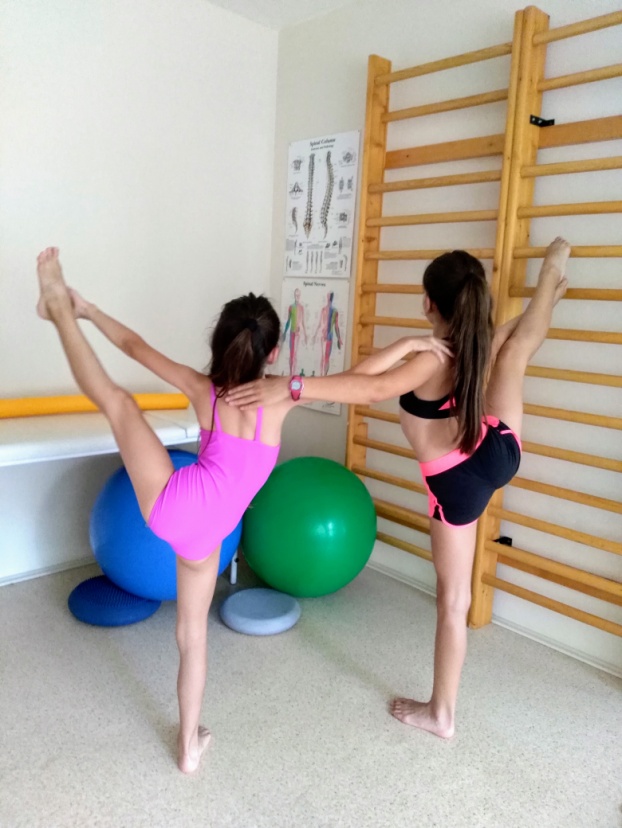 |
| --- | --- |
| 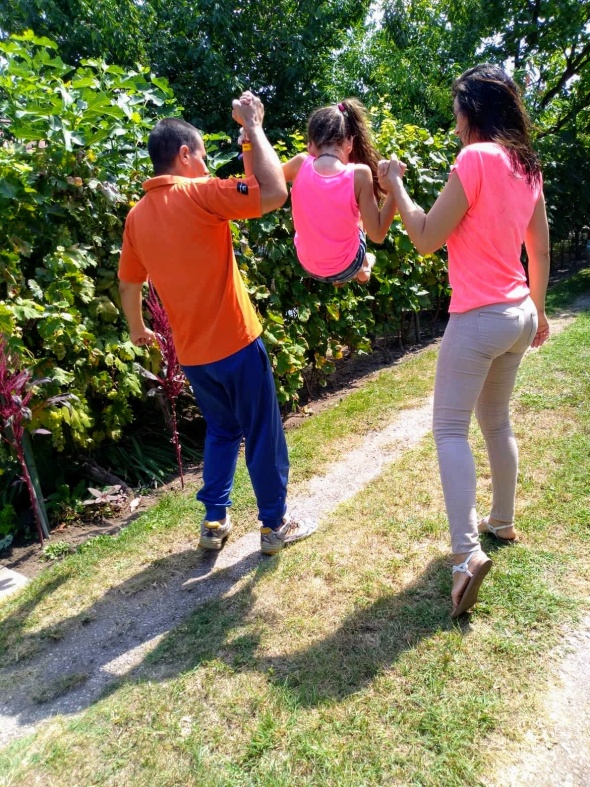 | 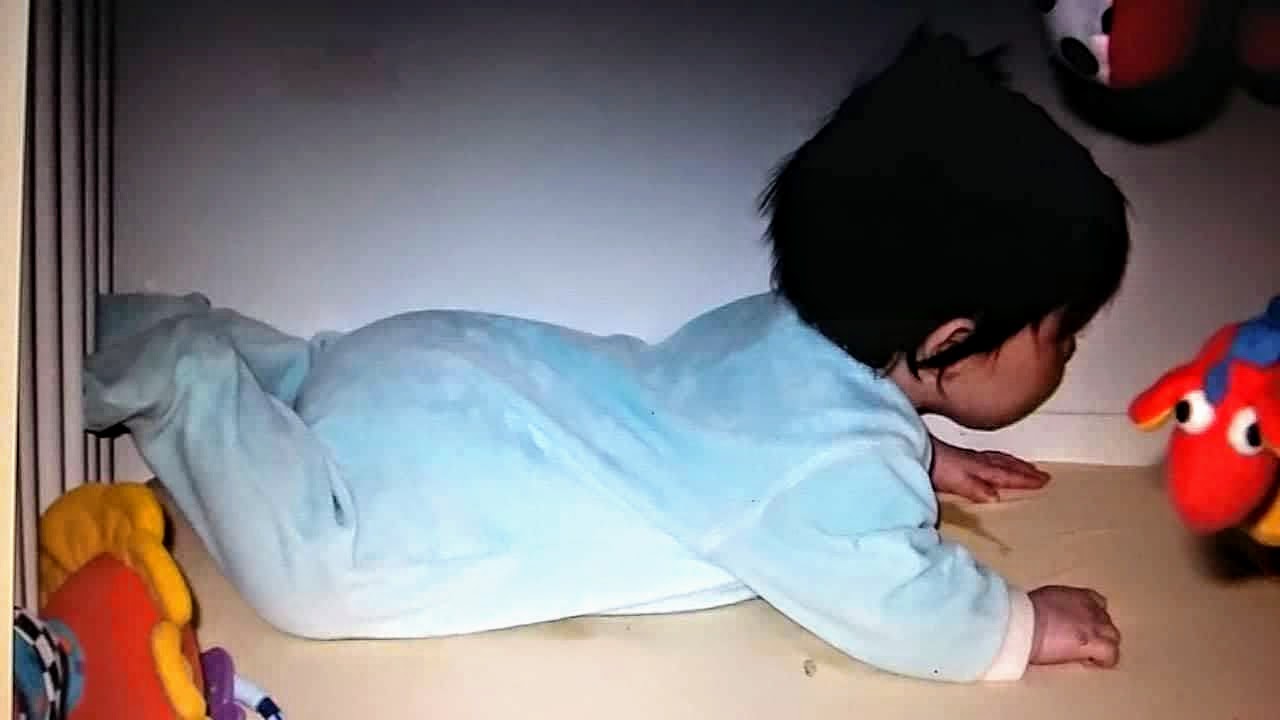 |

1. Color one vertebra to blue and one disc to red!


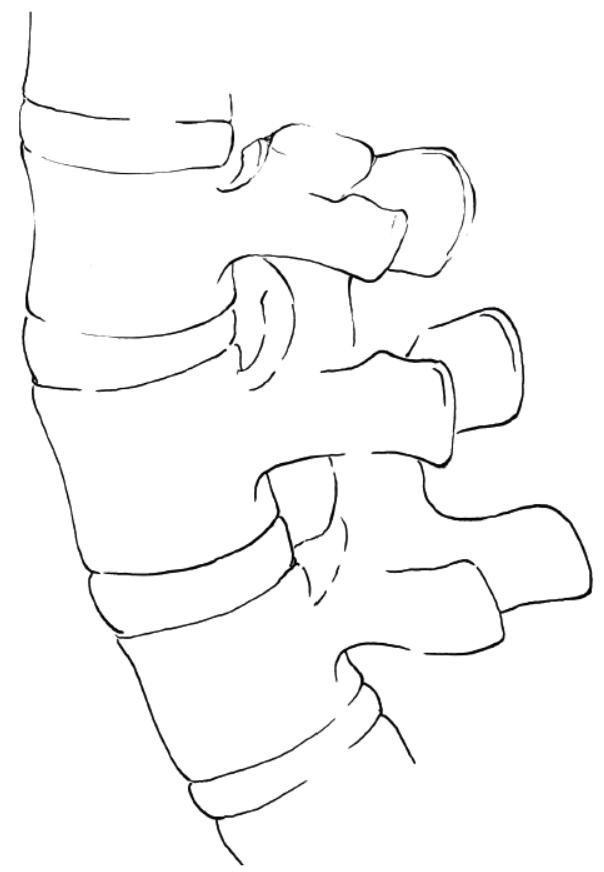


1. Mark 2 correct postures during watching TV!

| 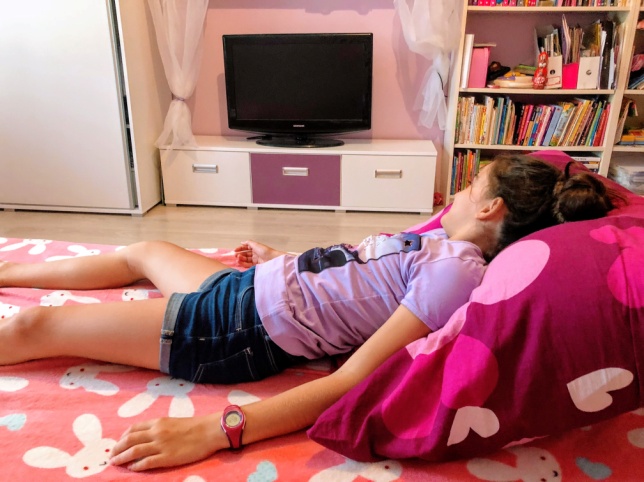 | 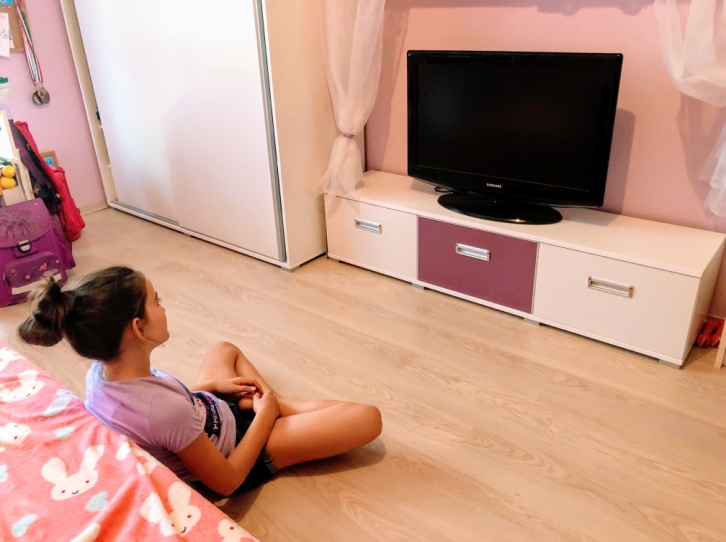 |
| --- | --- |
| 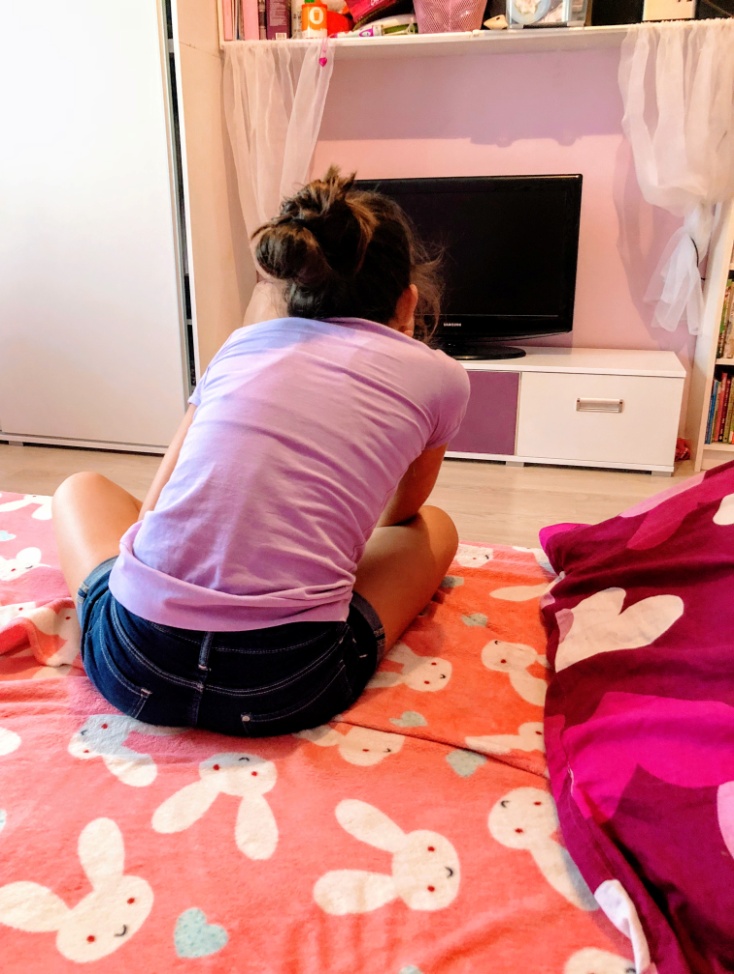 | 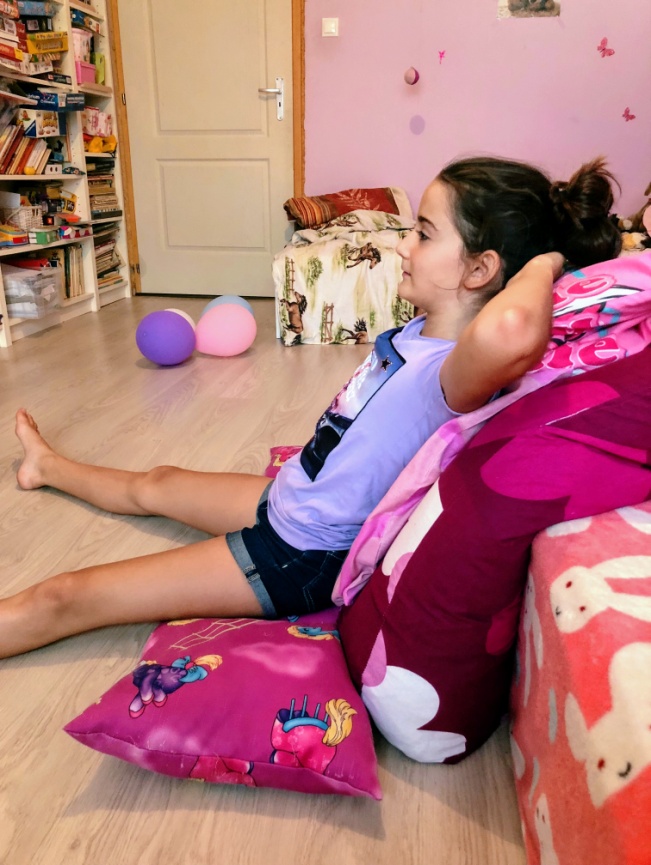 |
| 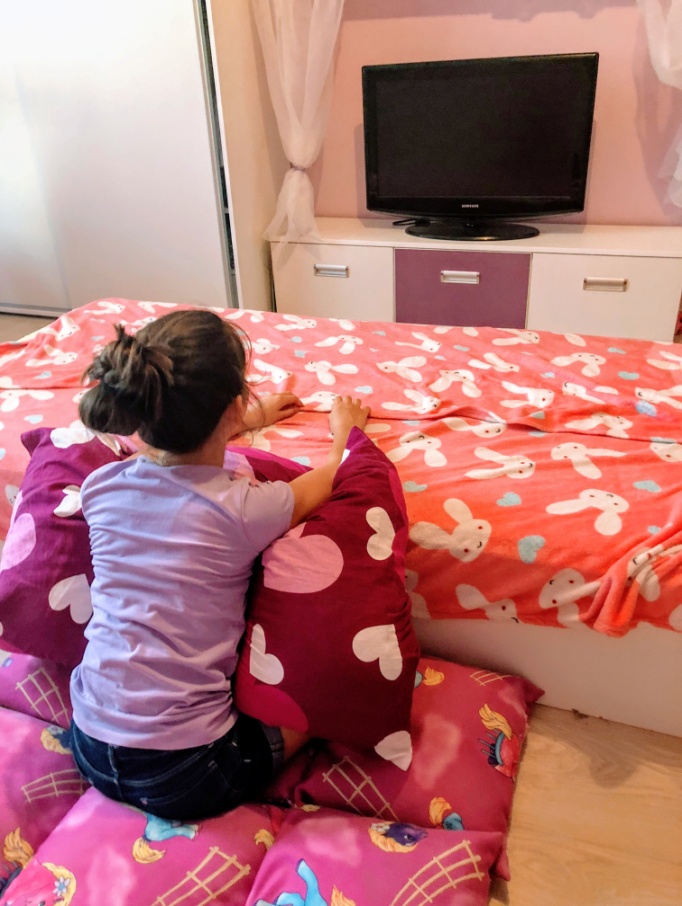 | |
|  | |

1. Mark 3 correct postures!

| 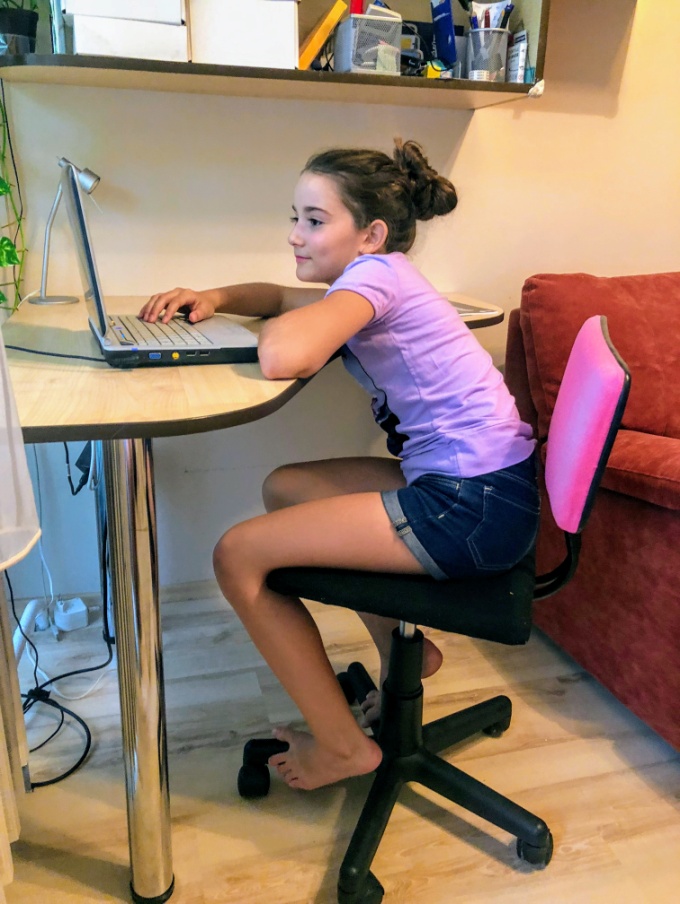 | 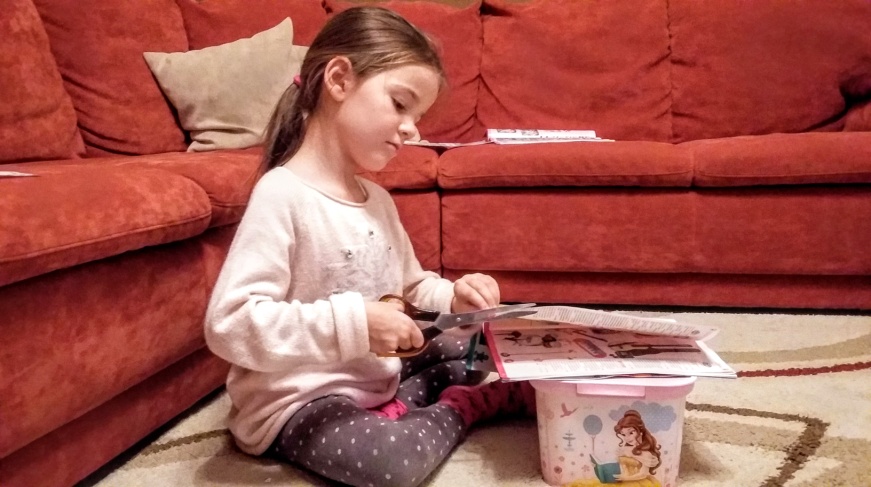 |
| --- | --- |
| 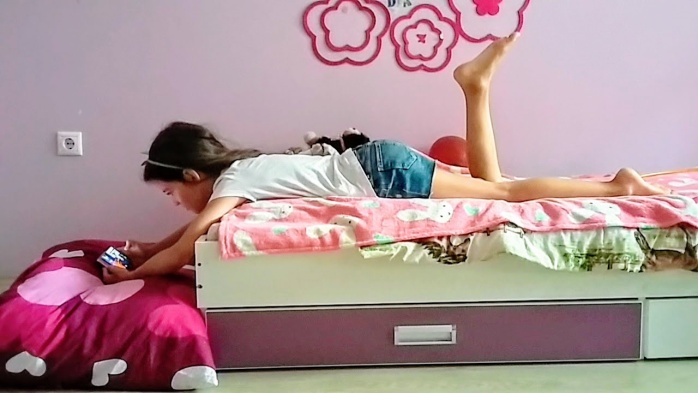 | 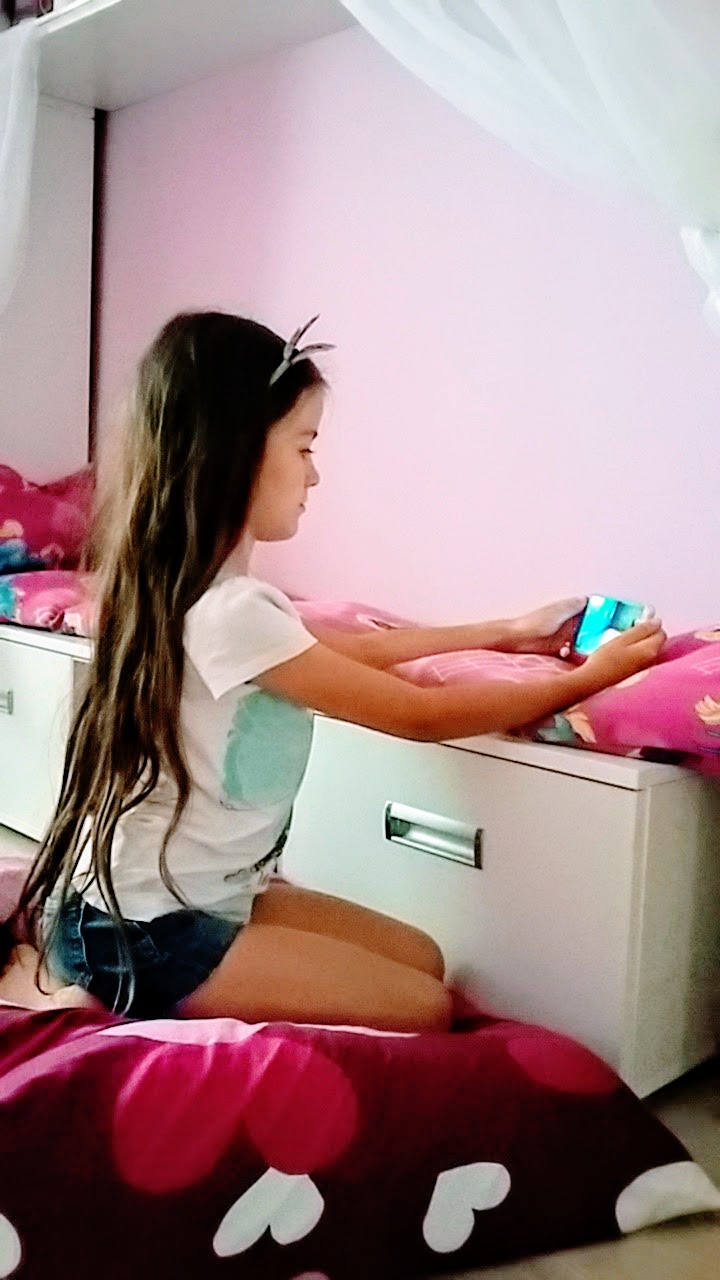 |
| 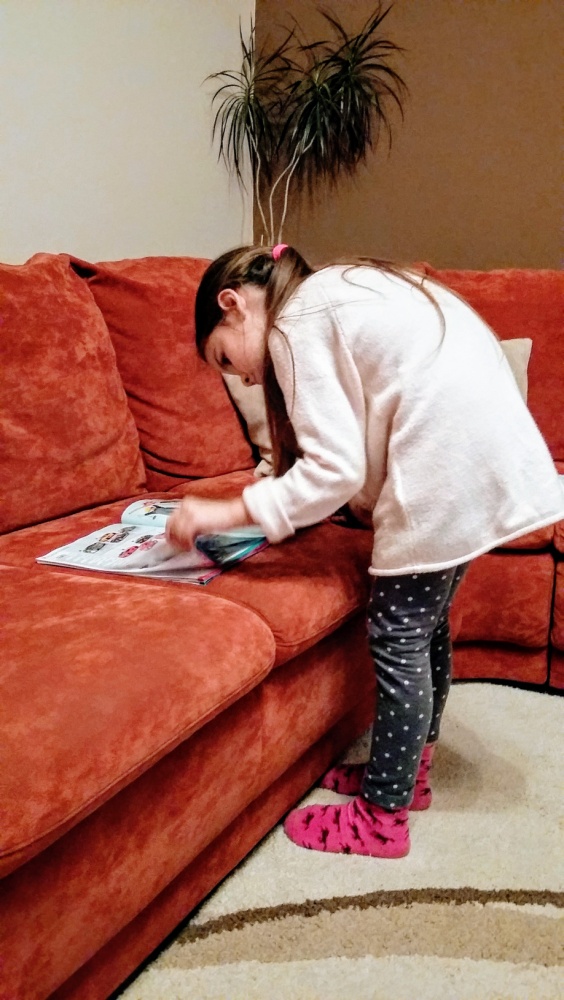 | 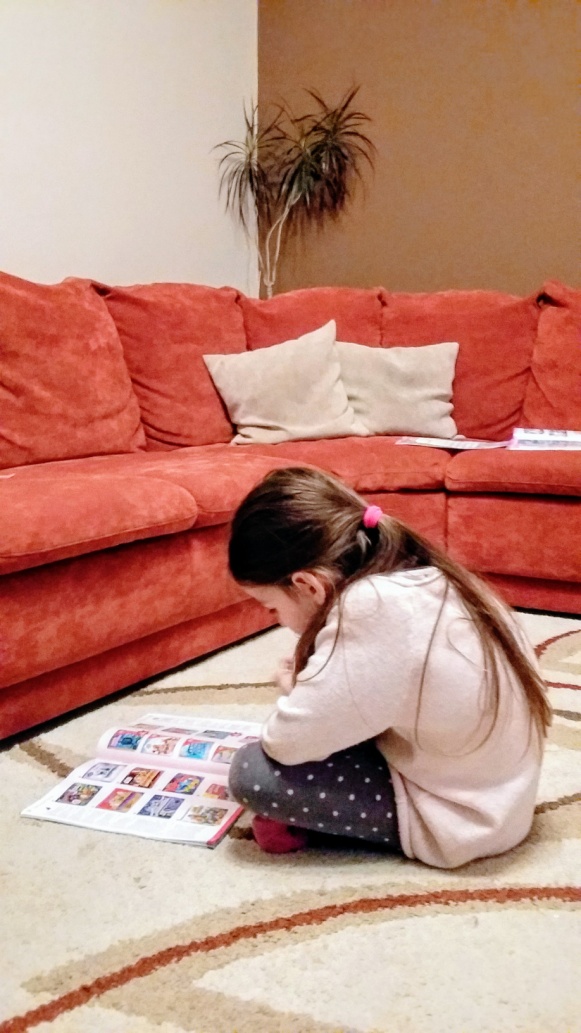 |
|  | |

1. Connect those with similar hardness!

| 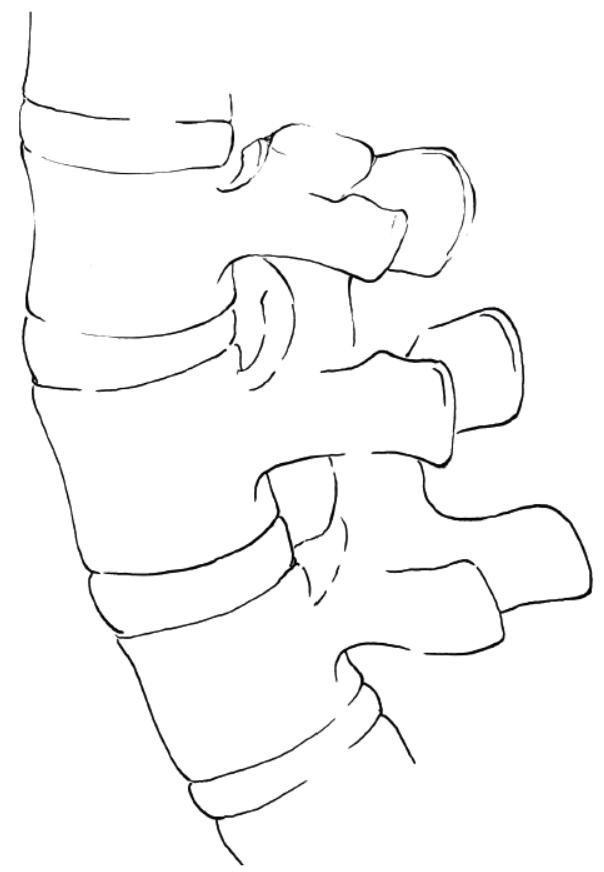 | 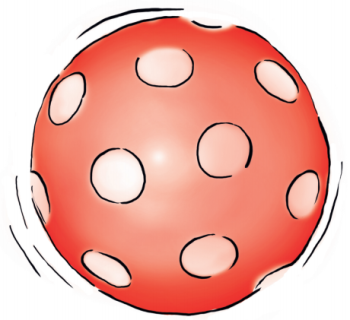 |
| --- | --- |
|  | 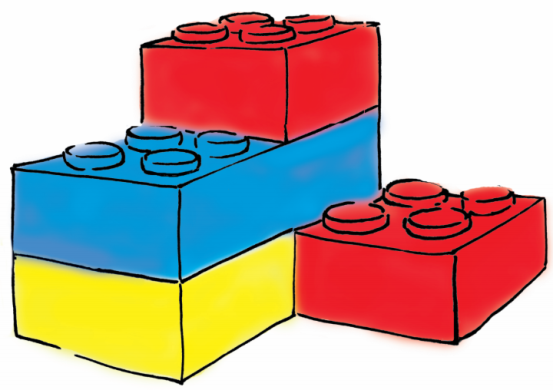 |
|  |  |

1. Mark, where the boy is correctly lifting the bag!


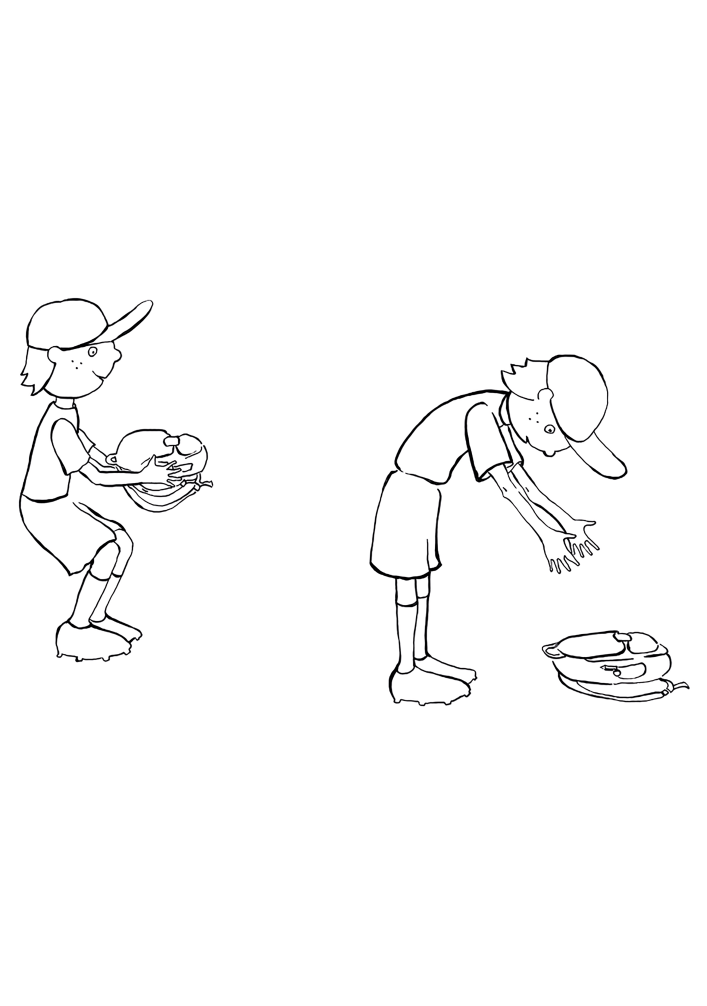


1. What holds and moves the spinal column?


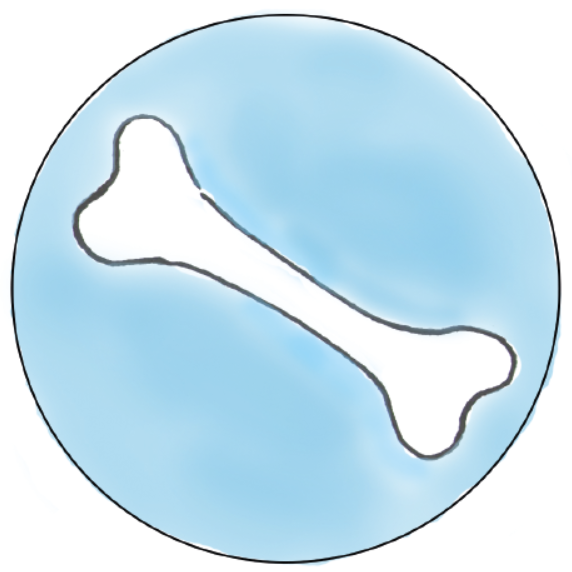

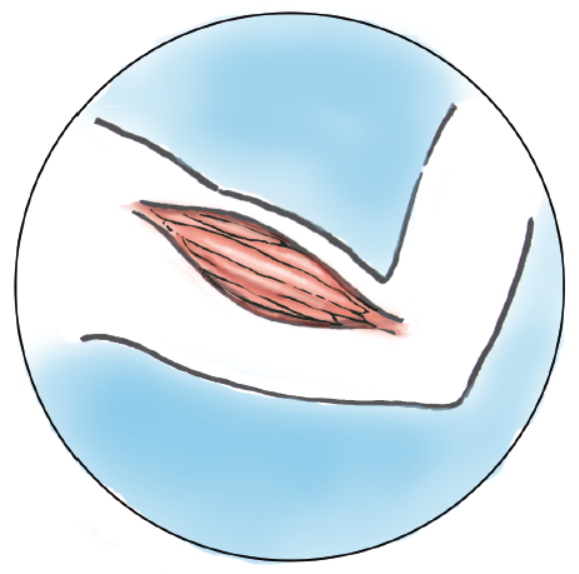

Supplement: Supplementary file 2 — Additional file 2. Health Questionnaire on Back Care Knowledge and Spine Disease Prevention for 6–10 Years Old Children. [file 12891_2021_4667_MOESM2_ESM.docx]
